# Nonlinear Fitness Landscape of a Molecular Pathway

**DOI:** 10.1371/journal.pgen.1002160

**Published:** 2011-07-21

**Authors:** Lilia Perfeito, Stéphane Ghozzi, Johannes Berg, Karin Schnetz, Michael Lässig

**Affiliations:** 1 Institut für Genetik, Universität zu Köln, Cologne, Germany; 2 Institut für Theoretische Physik, Universität zu Köln, Cologne, Germany; Université Paris Descartes, INSERM U1001, France

## Abstract

Genes are regulated because their expression involves a fitness cost to the organism. The production of proteins by transcription and translation is a well-known cost factor, but the enzymatic activity of the proteins produced can also reduce fitness, depending on the internal state and the environment of the cell. Here, we map the fitness costs of a key metabolic network, the lactose utilization pathway in *Escherichia coli*. We measure the growth of several regulatory *lac* operon mutants in different environments inducing expression of the *lac* genes. We find a strikingly nonlinear fitness landscape, which depends on the production rate and on the activity rate of the *lac* proteins. A simple fitness model of the *lac* pathway, based on elementary biophysical processes, predicts the growth rate of all observed strains. The nonlinearity of fitness is explained by a feedback loop: production and activity of the *lac* proteins reduce growth, but growth also affects the density of these molecules. This nonlinearity has important consequences for molecular function and evolution. It generates a cliff in the fitness landscape, beyond which populations cannot maintain growth. In viable populations, there is an expression barrier of the *lac* genes, which cannot be exceeded in any stationary growth process. Furthermore, the nonlinearity determines how the fitness of operon mutants depends on the inducer environment. We argue that fitness nonlinearities, expression barriers, and gene–environment interactions are generic features of fitness landscapes for metabolic pathways, and we discuss their implications for the evolution of regulation.

## Author Summary

The levels of protein produced by an organism are likely to change its fitness, potentially driving the evolution of genetic regulation. Importantly, protein expression generates costs as well as benefits. Here, we use a model genetic system, the *lac* operon of *Escherichia coli*, to investigate different sources of fitness costs. We find that fitness depends not only on the production rate of proteins but also on their enzymatic activity. A simple quantitative model, which is based on the biophysics of protein production and activity, accurately reproduces the experimental results and provides testable predictions. The model describes a feedback cycle between a molecular pathway and the growth rate of cells: pathway activity impedes growth, but growth itself affects the pathway. This feedback can generate dramatic effects, such as gene expression barriers, fitness cliffs, and population extinctions, which can be triggered by small environmental or genetic changes. Our results disentangle the complex interplay of protein production and activity, and they show how these processes shape the evolution of simple organisms.

## Introduction

Gene regulation is a major factor of molecular evolution, and changes in gene expression contribute to phenotypic differences between species [1]. Expression levels are under natural selection, which results from a balance between costs and benefits for the organism. For single-cell organisms, fitness benefits include the ability to digest nutrients in different environments. The cost of gene expression, on the other hand, depends on the biophysics of protein production and of protein activity. The cost of protein production has been studied extensively [2]–[6]. However, enzymatic activities of proteins can also reduce fitness due to energy consumption or toxic effects of the reaction products. What are the relative contributions of these two effects? How do they interact? To address these questions, we have to understand the fitness effects of an entire metabolic pathway, in which protein production is coupled to function and growth. This is the subject of the present paper.

For our analysis, we use the lactose utilization pathway in *Escherichia coli*, which is one of the best characterized molecular pathways [7]. It is coded in a set of genes referred to as the *lac* operon. Several studies have addressed fitness effects associated with expression of the *lac* genes. In particular, production of the *lac* proteins in the absence of lactose has been shown to involve a fitness cost, that is, to reduce the growth rate of a cell population [2]–[4], [8], [9]. This cost has been ascribed to transcription and translation of the *lac* genes [10], because toxic effects of the gene products have not been observed. Growth is also reduced by the presence of inducers in the medium, even after the maximum of expression is reached [8]. This fitness cost is likely to arise from inducer transport through the cell membrane [11], [12]. Furthermore, the *lac* operon has been used to study the interplay of cost and benefit in the evolution of gene expression [9]. Taken together, these observations make the *lac* operon an ideal system to study the coupled fitness costs of protein production and activity.

Here we determine a fitness landscape of the *lac* pathway by a combined experimental and theoretical approach. We measure the fitness of different regulatory mutant strains in the presence and absence of the *lac* inducer IPTG and of the natural sugar lactose. LacY proteins act as transporters (so-called permeases) for IPTG and lactose (i.e., these molecules are substrates of LacY). We develop a quantitative biophysical growth model to disentangle the fitness contributions of protein production (i.e., transcription and translation) and of protein activity (i.e., intra-cellular transport). The model explains the growth rate of all observed mutants in different inducer environments. Its key element is a *feedback loop* between the *lac* pathway and fitness: at constant rate of protein production, faster cell growth leads to stronger dilution of proteins and lowers the cost of protein activity. In addition, the rate of *lac* gene expression itself can depend on growth [13], [14]. Similar growth feedback mechanisms have been argued to play an important role in bacterial drug resistance [15], [16], and to generate diversity in an isogenic population [13], [15], [17].

Our analysis suggests that growth feedback is a pervasive feature of the activity-dependent fitness of metabolic pathways. This feature has important evolutionary consequences. In particular, our model predicts a *fitness cliff*, beyond which populations cannot maintain viable growth, and an *expression barrier*, that is, an upper bound for protein production and activity in viable populations. As a consequence, gene regulation in metabolic pathways is likely to be under stronger selection than the mere cost of protein production would suggest.

## Results

### Fitness effects of *lac* protein production and activity

**Figure 1:**
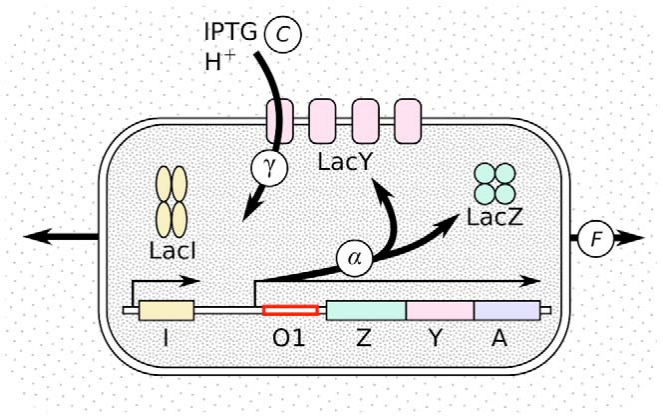
Schematic representation of the *lac* pathway. The *lac* operon is composed of three genes controlled by the same promoter: *lac* Z, *lac* Y, and *lac* A. The *lac* pathway also involves the constitutively expressed repressor LacI. It represses the transcription from the *lac* promoter by attaching to the operator sequence *lac*O1. Inducers, such as IPTG, deactivate the repressor LacI and thus stimulate the synthesis of the gene products LacZ, LacY, and LacA. The rate of production of the three *lac* proteins is denoted by 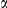 and it depends on the sequence of *lac* O1, on the presence of inducer inside the cell, and on the growth rate. All three *lac* genes are transcribed with the same rate, hence LacZ can be used as a reporter for the whole operon. LacY transports molecules such as IPTG inside the cell with a rate 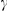, which depends on the concentration of these molecules. One proton 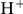 is transported with each substrate molecule [7]. Growth (measured by the Malthusian fitness 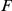) dilutes the internal molecules, thus lowering their concentrations. The strains used in this study differ by the *lac*O1 sequence and are grown in various IPTG concentrations.

There are two generic sources of fitness cost for a molecular pathway: the cost of protein production and the cost of enzymatic activity [2]–[6]. In the case of the *lac* pathway, fitness depends strongly on the presence of substrates of the *lac* proteins, even when these substrates cannot be used as a carbon source [8], [11], [12]. One such substrate is IPTG (isopropyl-1-thio-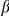-D-galactoside), which is transported by LacY and induces *lac* expression (see Figure 1). Hence, there are two potential phenotypes affecting the fitness of the *lac* operon in an IPTG environment: the rate of *lac* protein production and the rate of IPTG transport into the cell.

We measure the fitness effects of *lac* protein production and activity in thirteen regulatory mutants in the *lac* operon of *Escherichia coli*. Twelve mutant strains have substitutions in the *lac*O1 operator region, which affect expression of the *lac* genes, and one strain has a deletion in the gene of the repressor *lac*I (see Figure 1 and Text S1 for details). We determine the *lac* protein concentration and the fitness of these mutants both with and without substrates of the *lac* permease LacY. Specifically, we compete each mutant strain against a reference strain with deleted *lac* genes. This assay defines the fitness cost of the *lac* pathway as the difference in growth rate, or difference in Malthusian fitness, between reference strain and mutant, 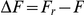 (see Materials and Methods and Text S1 for details).

**Figure 2:**
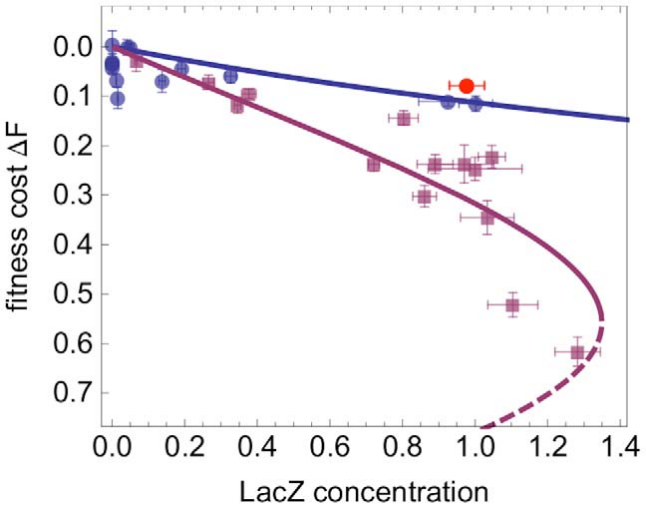
Fitness of *lac* regulatory mutants in different environments. Measured fitness cost of each mutant strain, plotted against LacZ concentration (normalized to the fully induced wild-type value). Measurements are obtained in minimal medium with 0.1% glycerol in the absence of IPTG (blue dots) and in the same medium with 1 mM IPTG (mauve squares). Fitness is measured by competition against a reference strain which has a deletion of the whole *lac* locus and of *lac* I 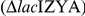. The fitness cost 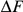 of a given strain is defined as the reduction in growth rate (Malthusian fitness) compared to the reference strain (see Materials and Methods for details). In presence of 1 mM IPTG, a control strain with deleted *lac* Y gene 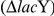 has an expression level comparable to the wild type, but a fitness close to that of constitutive mutants in absence of IPTG (red dot). All points show the average of 12 replicates for fitness and at least 3 replicates for protein concentration, with error bars giving the standard error. Lines show model predictions (the dashed line represents an unstable solution, see main text).

Figure 2 summarizes the results of these experiments. They show that fitness always decreases with increasing concentration of *lac* proteins inside the cell, but the form of this dependence depends on presence or absence of the substrate. Without substrate, the fitness cost can be fitted to a linear form, which we associate with *lac* protein production (blue line). When substrate is added, the magnitude of the fitness cost strongly increases and its dependence on concentration becomes nonlinear (purple line). The additional, nonlinear fitness cost in the presence of IPTG can be associated with the transport activity of the LacY proteins. This is shown by a control mutant with deleted *lac*Y gene, for which we only observe the linear cost of protein production (red dot). Deviations of individual data points from the fit curves can be caused by different sources of noise. Competition assays involve experimental errors, in particular for large fitness differences between strains. For example, there can be slight day-to-day differences in medium composition. Furthermore, some of the strains might have acquired mutations with a fitness effect outside the *lac* operator sequence, although we have controlled for random mutations elsewhere the genome (see Text S1).

As a further experimental step, we test whether these results extend to lactose, which is a natural nutrient of *E. coli*. The sugar used to support cell growth in the above experiments is glycerol, which is a poor carbon source. Lactose supports faster growth and is known to give an advantage to cells which are able to metabolize it. With 1 mM of lactose, the wild type has a *fitness benefit* over the reference strain, which amounts to 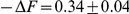 (mean of 4 replicates 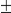 standard error). To assess whether lactose metabolism also involves a cost, we construct a mutant with deleted *lac*Z and *lac*I genes. This mutant cannot use lactose and expresses *lac*Y constitutively. In the presence of lactose, it has a *fitness cost* 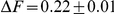 (mean of 12 replicates 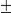 standard error) against the reference strain, which indicates that lactose and IPTG cause a similar decrease in fitness in the presence of the *lac* permease (see Materials and Methods for details).

We conclude that both the rate of protein production and the rate of protein activity (intra-cellular transport by LacY) are phenotypes that affect the fitness cost of the *lac* pathway. But what is the cause of the fitness nonlinearity in the presence of substrates, and what are its biological consequences? To address these questions, we now describe our experiments in terms of a simple biophysical model.

### Fitness model

We use a minimal model of gene expression and inducer transport to disentangle the fitness effects of protein production and activity in a quantitative way. The underlying intra-cellular processes involve transcription and translation, uptake of substrate by active transport, and dilution by cell division. Given the complexity of these processes and their effects on cell growth, our model does not aim at a complete description. However, the model does account for a large part of the fitness variation between strains and between cellular growth conditions. At the same time, it contains only few phenotypes and few parameters, which can be inferred from our fitness measurements.

Within the model, the cost of *lac* protein production is proportional the production rate 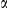, and we infer this rate from our measurements of fitness and LacZ concentration (see Figure 1 and Materials and Methods). The cost of LacY activity has two different potential contributions: the energy consumption of the transport process (direct transport costs) and growth effects of the molecules transported inside the cell (toxicity costs). Direct transport costs can arise from futile transport cycles: LacY transports one proton with every IPTG molecule, and ATP is consumed to pump the excess protons out of the cell. These costs are proportional to the total LacY pumping rate inside the cell, 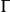. Toxicity costs are likely to arise from an excess concentration of the transported protons, i.e., a reduction of the intra-cellular pH value [11], [12]. The toxicity of IPTG itself appears to be negligible (see Text S1 and [12]). Toxicity costs are proportional to the steady-state concentration of the toxic molecules, which depends on their uptake rate, the rate of dilution by cell divisions, and the cell volume 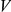 [18]. The excess concentration of protons is, thus, proportional to 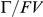. Furthermore, the steady-state cell volume itself depends on the growth rate, 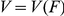 [13].

**Figure 3:**
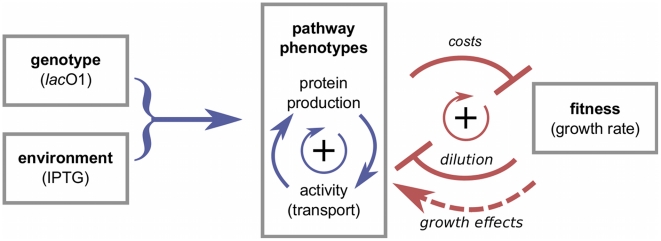
From genotype and environment to pathway phenotypes and fitness. Environment and genotype determine the function of the *lac* pathway, which is described by the two phenotypes of protein production and protein (transport) activity. These phenotypes are coupled by a pathway-specific positive feedback loop (blue circle). The pathway itself is coupled to growth (fitness) by a generic positive feedback loop: protein production and protein activity are fitness costs, and cell growth reduces protein concentration and activity by dilution (red circle). In addition, growth can affect the rate of gene expression [13] (dashed arrow). These feedback loops generate strong nonlinearities in the phenotype-fitness map and the genotype-environment-fitness map; see Figure 4 and Figure 5.

The combined fitness cost of protein production and activity in the *lac* pathway takes the form
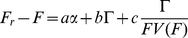
(1)in terms of the pathway phenotypes 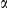 and 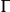. Here, 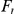 denotes the fitness of the reference strain with deleted *lac* genes (for which 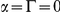). Our model contains a feedback loop: fitness depends on the rates 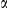 and 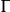, which in turn depend on fitness. This feedback between pathway phenotypes and fitness is illustrated in Figure 3. It has an important consequence: although the cost contributions in Equation 1 are taken to be additive at any given value of 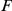, the resulting dependence of fitness on the pathway phenotypes, 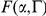, becomes nonlinear.

By calibrating this model to our experimental data, we can infer the amplitudes 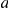, 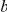, and 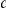 of the different cost factors. Bayesian analysis shows that there are significant fitness contributions of protein production and steady-state concentration (with maximum-likelihood parameter values 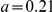, 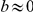, 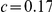), but the data are also compatible with a larger direct cost of transport (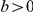) (see Materials and Methods and Text S1). As shown in Figure 2, the maximum-likelihood model provides a good fit to the data: the fitness feedback loop quantitatively explains the cost nonlinearity observed in our experiments.

**Figure 4:**
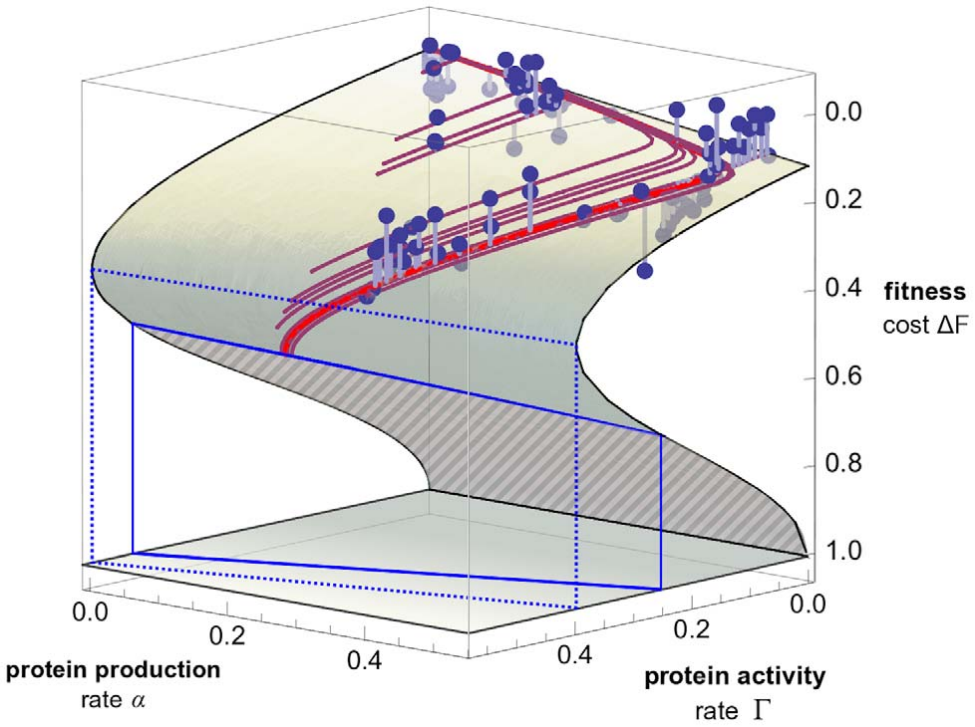
Phenotype-fitness map. The fitness cost 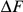 of the *lac* pathway is shown as a function of the protein production rate 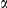 and the transport rate 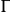. The fitness landscape obtained from our model (shaded surface) is strongly nonlinear and has two branches. The stable part of the landscape (solid shading) ends at a fitness cliff (solid blue line), beyond which populations cannot maintain growth. The remaining part of the lower fitness branch is unstable (striped shading). Protein expression and activity of viable populations are bounded by a barrier (dotted blue line). Model predictions of pathway phenotypes and fitness for individual strains under varying inducer concentrations are shown as a family of red lines (light red: wild type, dark red: operator mutant strains). Experimental fitness values are shown as dots (the offset from the model surface is marked by gray lines).

**Figure 5:**
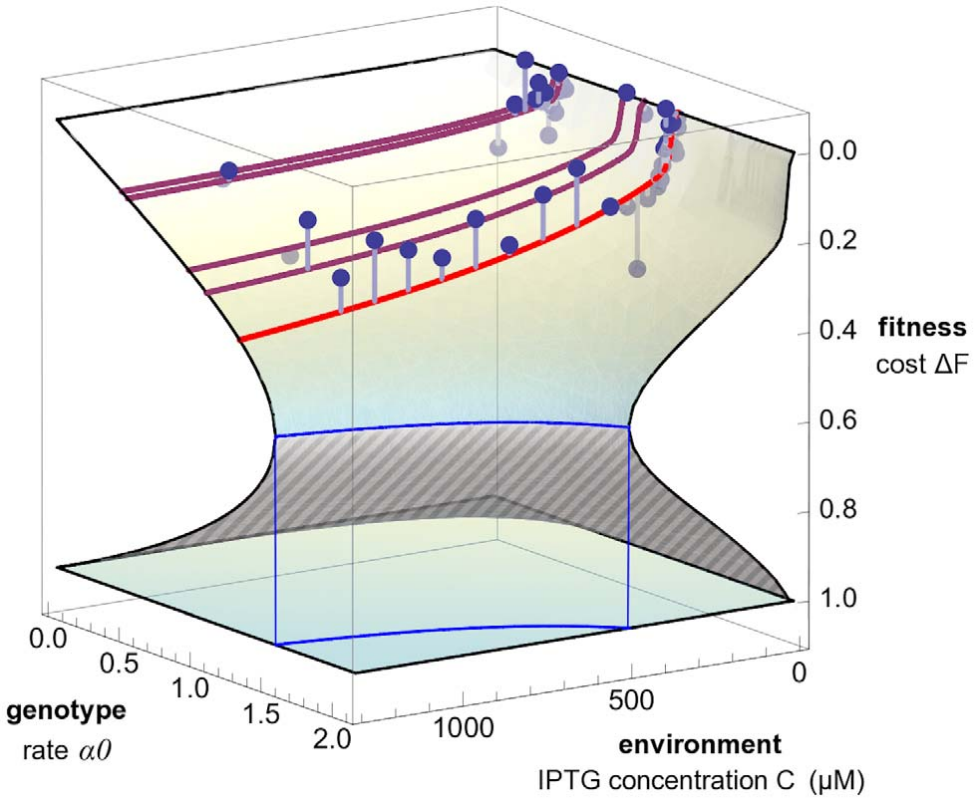
Genotype-environment-fitness map. The fitness cost 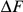 of the *lac* pathway is shown as a function of the operator genotype summary variable 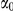 (maximum rate of protein production at a growth of 1 cell division/hr, see text) and the external inducer concentration 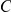. The model fitness landscape is again strongly nonlinear: it has a stable upper branch (solid shading) and an unstable lower branch (striped shading) separated by a fitness cliff (blue line), similar to the phenotype-fitness map of Figure 4. Model predictions for individual strains under varying inducer concentrations are shown as a family of red lines (light red: wild type; dark red: operator mutant strains with 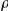 equal to wild type value, see text). Experimental fitness values are shown as dots (the offset from the model surface is marked by gray lines).

We use Equation 1 to derive two representations of a fitness landscape for the *lac* pathway, which highlight different biological implications of its form. First, we solve this equation to display the dependence of fitness on the pathway phenotypes, 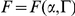, as shown in Figure 4. Second, we display the dependence of fitness on the external IPTG concentration, 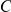, and on two genotype summary variables, which depend only on *lac* O1 sequence. As genotype variables, we use the maximal rate of *lac* protein production at a fixed growth rate of one cell division per hour, 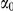, and the ratio of repressed to unrepressed protein production rates, 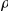 [19]. The resulting function 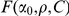, which is shown in Figure 5, can be called a genotype-environment-fitness map. We note that the change from the phenotype variables 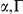 to the genotype-environment variables 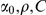 depends itself on fitness. This dependence has two reasons: (i) The LacY pumping rate depends on the production rate, the pumping rate per LacY molecule 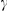, and fitness, 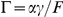, because LacY molecules are diluted by cell divisions just like the transported molecules. This generic dependence reinforces the basic growth feedback loop by dilution, which also enters Equation 1. (ii) For fixed genotype and environment, the production rate itself can depend on fitness, 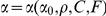. This growth effect on gene expression generates an additional feedback between the *lac* pathway and fitness, which is expected under several growth conditions [13], [14]. Including this feedback in our model significantly improves the agreement between data and theory (see Materials and Methods and Text S1 for details). The fitness landscapes of Figure 4 and Figure 5 are obtained from our model using maximum-likelihood parameters, but their shape depends only on the presence of a fitness nonlinearity (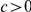). We now discuss their form and their biological implications in more detail.

### Phenotype-fitness map

The phenotype-fitness landscape of the *lac* pathway resulting from our model is shown in Figure 4, together with fitness measurements of different *lac* O1 operator mutants in different inducer environments. The experimental data are plotted as a function of the pathway phenotypes 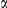 and 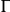 inferred from our model; for each mutant, the dependence of these phenotypes on the IPTG concentration is indicated by a red line. Data and model consistently show that protein production and activity of the *lac* pathway affect fitness in a highly nonlinear way. Our model explains the nonlinearity in terms of the growth feedback mechanism contained in Equation 1.

This form of the phenotype-fitness landscape has two important aspects. First, the nonlinearity of fitness translates into epistatic interactions between the pathway phenotypes: the effect of a change in the production rate 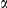, which is proportional to the slope 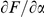, depends on the transport rate 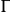, and vice versa. Second, the fitness landscape is not univalued: for some values of 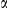 and 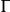, there are two possible fitness values, for others, there is none. Phenotype values in the no-solution regime cannot be attained by a cell population in steady growth. This regime is bounded by a dotted line in the 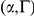 plane, which marks an expression barrier for the *lac* genes. The barrier occurs at a finite growth rate 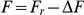 (in contrast to the model of ref. [9]). Double-valued fitness solutions and the existence of an expression barrier for given phenotype values are a direct consequence of the growth feedback loop in Equation 1. The stability analysis described below shows that only the full-shaded part of the landscape describes viable cell populations in stationary growth, whereas the striped part is unstable. Hence, for parameter values between the dotted and the solid lines in the 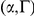 plane, populations can reach two different steady-state growth rates with the same *lac* pathway phenotypes.

### Genotype–environment interactions

We now turn to the dependence of fitness on the *lac* O1 operator sequence and on the external inducer concentration 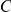, which are the quantities we manipulate in our experiments. To display the sequence-dependence, we use the genotype summary variables 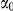 (maximal rate of *lac* protein production at a fixed growth rate of one cell division per hour) and 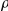 (ratio of repressed to unrepressed protein production rates). These variables reflect the double role of the operator sequence: it acts as a binding site for the repressor LacI, but it also affects other processes that lead to changes in protein production [20]. Figure 5 shows the fitness cost as a function of the maximal production rate and the IPTG concentration, 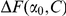. The ratio 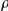 is kept fixed to its wild type value; the figure shows fitness data for the corresponding subset of strains (see Figure S1 and Figure S2 for the full dependence of 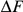 on 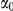, 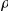 and 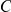). Again, cell populations in stationary growth cannot exist for some genotype-environment parameters; this regime is bounded by a blue line in the 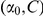 plane.

The fitness of different mutants as a function of the inducer concentration is again shown as a family of lines. The nonlinearity in the landscape indicates that the inducer environment affects the selective effect of regulatory mutations: higher IPTG concentrations lead to increased fitness differences between mutants. This interaction between genotype and the environment is due to an increase in the pumping rate with increasing IPTG, to the coupling of uptake rate and production rate in the term 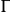, and to the growth feedback through dilution. Figure S2 further illustrates this interaction. Genotype-environment interactions in the *lac* operon have been observed previously [21]. Our model shows how such interactions emerge from the basic architecture of metabolic pathways.

### Nonlinearities generate extinction thresholds

The fitness landscapes of Figure 4 and Figure 5 have a common feature: over a wide range of parameters, there are two possible fitness values 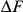. This double-valued fitness landscape is partitioned into a stable part (full-shaded) and an unstable part (striped); see Text S1, Figure S3, and Figure S4. The stable part of the landscape describes stationary growth of viable populations; i.e., cells with growth rates close to a point on this surface reach a steady state given by a point on the surface. A large part of the lower surface 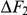 is unstable, i.e., cells with fitness cost 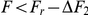 are unable to dilute their proteins and transported molecules fast enough to maintain stable growth. These cells will further decline in fitness, whereas cells with 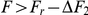 will increase fitness up to the stable value 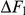. The stable and the unstable part of the fitness landscape are separated by a fitness cliff, which is shown as a blue line in Figure 4 and 5. The cliff marks an extinction threshold: If a cell population is driven beyond this cliff by mutations or environment changes, it suffers a sudden drop in fitness and cannot maintain a finite growth rate.

Existence and position of the fitness cliff depend on the amount of inducer present (see Figure 2, Figure 5, and Figure S2). For IPTG concentrations used in our and other experiments, the cliff is far from the wild type (Figure S2B). We note, however, that lactose is often used in higher concentrations, and lack of growth due to the presence of lactose (lactose killing) has indeed been observed [22].

## Discussion

We have shown that in the presence of an inducer, the fitness cost of the *lac* pathway arises not only from protein production, but also from transport activity of the permease LacY. The cost is governed by a feedback loop, which is the result of two repressive interactions: protein activity results in reduced growth, and growth dilutes proteins as well as transported molecules (see Figure 3). We note that our feedback mechanism does not rely on a limitation of cellular resources to generate a nonlinear relation between *lac* gene expression and growth (in contrast to the model of ref. [9]). This feedback produces a strongly nonlinear dependence of fitness on pathway phenotypes or on genotype and environment, as shown in the fitness landscapes of Figure 4 and Figure 5. Both landscapes contain a fitness cliff, which is an extinction threshold for cell populations. The nonlinearity of fitness is likely to persist for any substrate of the permease LacY and sets an upper bound for its rates of expression and activity. Thus, changes in *lac* permease activity or expression can have strong impacts on fitness. This is consistent with the observation that *lac*Y is under particularly strong selection [23], as reflected notably by its low number of synonymous single-nucleotide polymorphisms [24].

The nonlinearity of fitness and its consequences are expected to hold in the presence of lactose. If the benefit conferred by lactose (or other sugars) also depends on its internal concentration, we expect an effect of diminishing return: the faster a cell grows, the more it will dilute lactose, which leads to a sublinear increase of fitness with lactose concentration. Hence, combining costs and benefits of the *lac* proteins will lead to more complex fitness landscapes; their detailed dependence on pathway phenotypes will be addressed in a future study. Importantly, the full landscapes are expected to have a fitness cliff similar to the cost landscapes derived in this paper. This might explain why induced cells grown in a chemostat die after exposure to high concentrations of lactose, a phenomenon known as lactose killing [22]. Moreover, many other metabolic pathways in microorganisms contain a membrane pump or transporter accumulating substrates inside the cell, which often uses the proton motive force as an energy source. Our results are expected to apply to these pathways as well. In particular, we note the similarity of our fitness landscapes and those of the glucose utilization pathway in yeast [25] (see Figure 5 and Figure S2). Other protein activities such as hydrolysis of substrates can produce the same type of feedback, because they also depend on internal concentrations of molecules.

The shape of the fitness landscape described here has various implications for the genomic evolution of the *lac* pathway. Our fitness model of protein production and activity contains two types of epistasis on the operator *lac* O1. Within the operator, the fitness reduction caused by two mutations that increase expression is larger than the sum of the fitness costs of either one (see Figure 5). Furthermore, the selection pressure on expression depends on the protein activity rate and, hence, on the sequence of the downstream gene *lac* Y. The total pumping rate of the cell also depends on the concentration of LacY substrates in the environment, which generates fitness interactions between the operator genotype and the inducer environment. In a broader context, the costs of gene expression due to protein activity and due to protein production affect the evolution of regulatory systems in a different way. Taking into account only protein synthesis, we expect the length of genes to be the main determinant of the fitness cost of gene expression. Including protein activity, however, the selective pressure against expression of a gene can depend primarily on the coding sequence of functional domains and on the environment. For the *lac* pathway, the cost contributions of protein production and of protein activity are of similar magnitude, and both effects contribute to selection on regulatory sequences.

Generalizing the results of this study, we expect the full landscape of a metabolic network to be filled with cliffs and valleys, whose importance depends on which pathways are more active in a given environment. In addition, a metabolic pathway with growth feedback generates ubiquitous epistasis. For example, any mutation under selection has fitness interactions with mutations in the *lac* operon: In the presence of IPTG, deleterious (beneficial) mutations outside the *lac* pathway affect the protein production rate 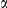 and the transport rate 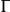, and hence increase (reduce) the fitness cost of *lac* activity-enhancing mutations. Thus, higher-dimensional fitness landscapes including more and more metabolic phenotypes are expected to be increasingly rugged.

Previous experiments have produced fitness landscapes as a function of genotype (see for example [26], [27]). This kind of fitness landscapes omits the intermediate level of phenotypes, which describes how genotype changes affect biophysical functions. Here, we record fitness as a function of well-defined phenotypes of a metabolic pathway. These can be connected to a biophysical model, which describes the dependence of fitness on the operator sequence and on the inducer concentration. Phenotype- and model-based fitness landscapes are predictive: Once the model constants are fixed by one set of measurements, the model predicts the outcome of further experiments with different input parameters. In this study, the most striking model prediction is the extinction of populations beyond a fitness cliff.

Our fitness landscape also differs from previous phenotype-fitness maps, perhaps the most popular of which is Fisher's geometric model [28]. Fitting this model to fitness data is a method to infer distributions of fitness effects of mutations and of epistasic effects between mutations [29], [30]. Fisher's geometric model contains an *a priori* arbitrary number of unknown molecular phenotypes. In contrast, our model contains a small number of known phenotypes associated to a specific pathway, which are shown to capture salient features of fitness variation between populations (clearly, this does not rule out further phenotypes of this pathway affecting fitness). In the classical geometric model, the fitness landscape is assumed to be smooth, and different phenotypes to contribute additively to fitness. Our fitness landscape contradicts both of these assumptions: there is strong epistasis and ruggedness. These features have been extensively analyzed for genotype-fitness maps (a well-known example is the NK model [31]), but the dependence of fitness on quantitative phenotypes is generally assumed to be smoother. Our study shows that strong epistasis and ruggedness can persist in phenotype-fitness landscapes. It calls for new statistical models of such landscapes, which address their broad consequences for speed and constraints of molecular evolution. An interesting example is a recent extension of the geometric model, which contains epistasis and a fitness cliff [32].

In summary, our measurements and modeling show that the *lac* pathway of *E. coli* is governed by a strongly nonlinear fitness landscape depending on phenotypes of protein production and activity. These phenotypes, in turn, depend on the *lac* operon genotype and on environmental parameters in a coupled way. Fitness nonlinearities and genotype-environment interactions are not specific to the system studied here, but are likely to be general features of metabolic pathways. Thus, the fitness landscape of a metabolic network is much more than a simple superposition of the cost of protein production and the benefit of protein activity. It describes the entire network as a unit of natural selection. Such system-level fitness landscapes emerge already at simplest level of cell growth and metabolism.

## Materials and Methods

### Strain construction

The background of all strains used in this study is *Escherichia coli* BW30270 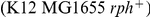. The *lac*O1 mutant strains (summarized in Table S1 and Table S2) are constructed as described in [33]. First, the complete *lac* promoter is deleted and replaced with the chloramphenicol resistance cassette from plasmid pKD3 (see Table S3 for a list of plasmids used in this study). This yields strain S4146 which is 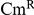, 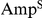 and 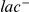. The full *lac* promoter and 5′UTR of wild-type *Escherichia coli* are amplified and cloned (see Table S4 for a list of oligonucleotides used in this study). Specific *lac* O1 mutations are inserted using PCR mediated mutagenesis [34], and the mutant sequences are cloned in a high-copy-number plasmid (derived from pUC12). The same gene replacement method [33] is then used to replace the chloramphenicol resistance cassette in strain S4146 with the chosen *lac* promoter and O1 operator. The strains produced in this way are all 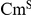, 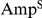 and 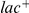. We noticed that these strains have a general lower fitness than strain BW30270 that cannot be explained by the inserted mutations (see Figure S5) so we use T4GT7 mediated transduction [35] to transfer the *lac* mutations back to the parent background (BW30270). First, the resistance cassette from strain S4146 is transduced to BW30270, producing strain T218. Then, the mutated *lac* operon is transduced from each *lac*O1 mutant to T218. The *lac* promoter and O1 operator are then sequenced to confirm the correct insertion of the *lac* operator allele. As a control for the transduction, a wild type construct is obtained in the same way (T273). It has the same fitness as BW30270. The reference strain for the competition 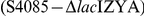, the *lac* permease and the *lac* repressor mutants (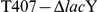 and 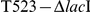) are constructed as described in [33]. Strain 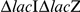 is constructed by first deleting *lac* Z following [36] and then deleting *lac* I following [33].

### Media and growth conditions

Unless stated otherwise, all measurements are made in M9 minimal medium with glycerol (0.1% v/v) as carbon source. To distinguish strains in competition, tetrazolium lactose (TL) medium (1% bacto-tryptone, 0.1% yeast extract, 0.5% NaCl, 1% lactose, 0.005% tetrazolium chloride and 1.5% agar) is used. 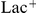 colonies are white and 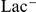 colonies are red in TL plates [37]. 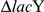 is also white on TL plates. To distinguish this strain from 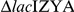 and 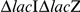, LB-XGal-IPTG plates are used (1% bacto-tryptone, 0.5% yeast extract, 0.5% NaCl, 1 mM isopropyl-1-thio-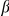-D-galactoside (IPTG), 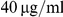 5-Bromo-4-chloro-3-indolyl-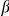-D-galactoside (X-Gal) and 1.5% agar).

### Protein expression

Protein concentration is estimated using a 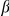-galactosidase (LacZ) activity assay [38]. Since all our mutants have the same coding sequence for this protein, changes in activity reflect changes in protein concentration inside the cell. The LacZ assays are performed as described in [38]. Overnight cultures are diluted in fresh medium to an optical density at 600 nm (OD600) of 0.05 and harvested after growth in the indicated media at 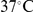 to an OD600 of 0.3. IPTG was added to the overnight culture and to the test cultures in the concentrations mentioned in the text. The enzyme activities are determined from at least three independent cultures. Figure S6 shows the measured LacZ levels for all strains used in this study, in absence and presence of IPTG.

### Fitness measurements

Fitness is measured in head to head competition as described in [39]. Briefly, frozen cultures (stored at 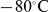) are streaked on a Luria broth agar plate and grown over night at 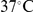. An isolated colony is randomly selected and grown overnight in 3 ml of the same medium used in the competition, in particular with the same amount of IPTG. Both the reference strain (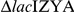, unless stated otherwise) and assay strains are treated in this way separately. The strains are then mixed and diluted in saline solution (10 mM 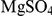 and 0.85% NaCl), such that about 50,000 colony forming units (CFUs) of each strain are used to start the competition. The mixed dilutions are also used to count the starting titer. Cultures are grown for 24 h on 96 deep-well plates in 1 ml of medium, shaken at 150 RPM, reaching saturation (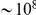 CFUs). They are then diluted and plated on TL or LB-XGal-IPTG medium.

We measure the Malthusian fitness 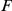, i.e., the growth rate, of each strain in units of the growth rate of the reference strain (such that 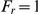). The fitness value of a mutant is inferred from a competition experiment with the reference strain,
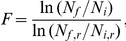
where 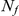, 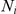 are the final and initial number of mutant CFUs after and before the competition, and 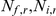 are the corresponding numbers for the reference strain. The growth rate of the reference strain is not affected by IPTG (see Figure S7). Thus, the doubling time of the reference strain is a fixed time unit and fitness measurements across environments are directly comparable. We report the fitness cost of a mutant compared to the reference strain, 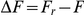 (which is proportional to its selection coefficient measured in units of doubling time, 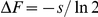 [40]).

The 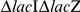 strain has the same phenotype as the reference strain (both are red on TL plates and white on LB-XGal-IPTG plates), so the two cannot be competed directly. Instead, we measure the fitness of this strain by competing it with 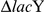. 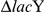 has the same fitness as the reference strain in competition in glycerol minimal medium with 1 mM lactose (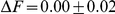).

### Dependence of pathway phenotypes on genotype and environment

As explained in the Results section, the protein production rate 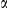 and the transport rate 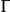 can be expressed in terms of genotypic and environmental parameters, and fitness. This map relates the fitness landscapes of Figure 4 and Figure 5 and can be obtained as follows.

The first phenotype of the *lac* pathway, the protein production rate 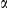, has two main components: one is independent of the lac repressor (LacI) and the other depends on the probability of the repressor to bind the operator. The independent component is given by the direct effect of the operator sequence (quantified by the first genetic component 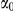) and by the growth rate 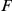 (through a function 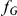 specified below). The LacI-dependent component of 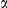 depends on the affinity of the operator sequence (measured by the second genetic component 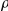) and on the concentration of inducer 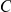 in the environment. The dependence on 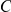 has the form of a Hill function 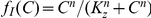 with parameters 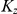 (the half saturation constant, taken to be 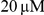) and 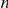 (the Hill coefficient, taken to be 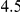) [18]. The protein production rate is then 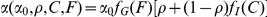. We now derive the form of 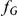, and estimate 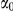 and 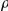.

As mentioned before, 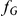 is the dependence of the production rate on the growth rate 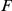. Following [13], [14], 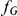 is expressed relative to the fitness of a strain growing at the rate of 1 doubling/hour, 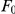, such that 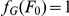. Note that the reference strain has a growth rate of 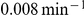, so 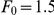. The parameter 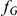 reflects the following observation: When the growth rate changes due to nutrient quality, there is a linear inverse correlation between protein concentration (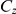) and growth rate [14], 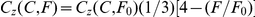 (see Figure S8A). This relationship can be extended to the protein production rate 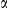, because 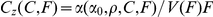 at steady state. We choose a linear dependence of the cell volume 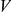 on the fitness, 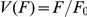; see Text S1 and Figure S8B. Using the dependences inferred above and assuming 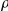 to be independent of 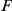, the dependence of protein production on growth rate can be estimated: 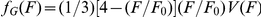. We have verified that including 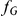 significantly improves the agreement between model and data (see Text S1), although it is not obvious *a priori* that a correlation between 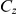 and growth rate is relevant in the context of our experiments.

The two genetic components, 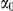 (the maximal protein production rate at fixed growth rate) and 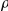 (the ratio of repressed to unrepressed protein production rates), depend only on the genotype and were calculated for each strain separately. 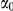 can be derived from the protein concentration and fitness measured at a concentration 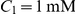 IPTG, where the LacI proteins cannot bind DNA (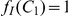). As explained above, the cell volume 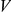, the growth rate 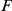, and the effects of growth on expression 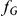 affect 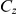, such that 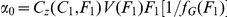, where 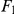 is the measured growth rate at 1 mM of IPTG. Similarly, 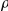 can be estimated using 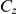 and 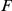 measured at 0 mM of IPTG: 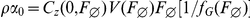, where 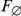 is fitness in the absence of IPTG. Both 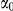 and 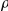 are independent of the model in Equation 1 and of the growth-dependence of the volume. Inferred values of 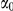 and 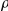 are shown in Figure S9 and Table S1. The parameter 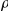 is related to the “repression level” 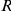 defined by Müller-Hill and co-workers as the ratio of LacZ activity between strains differing only by the presence/absence of the *lac* repressor, 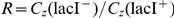 [19]. Neglecting the growth difference between both strains, these quantities are inversely related, 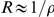.

The second phenotype of the *lac* pathway, the total transport rate 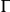, is the product of the number of LacY molecules in the cell and the transport rate per LacY molecule, 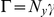. The number 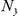 is equal to 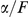, with 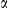 the protein production rate and 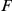 the growth rate, because LacY molecules are diluted by cell divisions. Note that 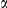 is measured for LacZ, but all proteins of the operon are produced proportionally. The ratio of LacY molecules per LacZ molecule, which is close to 3 [10], and other numerical constants are absorbed in the coefficients 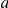, 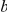 and 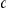. The transport rate 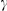 depends on the external IPTG concentration, 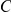, and on the half-saturation constant for inducer uptake, 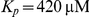 [41]. An expression for 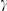 can be derived from the known functioning of the permease [42], with efflux neglected (see Text S1). We obtain 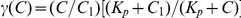, normalizing 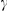 to 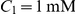 of IPTG.

The uncertainties on 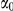 and 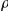 are obtained by standard error propagation, assuming independent experimental errors on 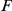 and 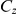 (see Text S1). A possible error in the IPTG concentration 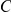 is not considered, because it is expected to be small.

The coefficients 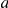, 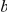 and 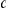 in Equation 1 are obtained by likelihood analysis of our model and the experimental data. This analysis is based on the dependence 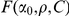, where 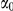 and 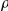 are inferred for each mutant as described above. The fitting procedure and score-based model comparisons are detailed in Text S1 (see also Figure S10 and Figure S11).

## Supporting Information

Figure S1Fitness cost 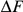 as a function of the IPTG concentration for five strains: (A) the wild type, (B) T274, (C) T320, (D) 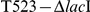, (E) T275. The full lines are model predictions; dots show the experimental data (the error bars represent the standard error of the mean on 4 replicates, on 12 replicates for data at 0 and 1 mM IPTG). See Table S1 for a list of strains and the corresponding values of 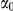 and 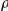.(TIF)

Figure S2Fitness cost 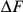 as a function of 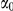 and 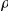, at fixed external IPTG concentration (A) 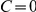, (B) 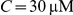 and (C) 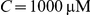; as a function of 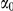 and 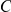, at fixed (D) 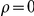, (E) 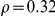 and (F) 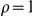. The dots are the experimental data, the grey vertical bars show the distance between data and model prediction. Strains shown: (A) and (C) all operator mutants, wild type, 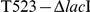 (D) wild type, T319, T320, T378, T379; (B) wild type, T274, T275, T320, 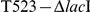; (E) T323; (F) T275, 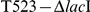. The light-green surfaces show the stable solutions, the dark gray the unstable one. The blue line marks their boundary: when 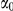 or the external IPTG concentration is increased beyond this “cliff”, the population falls on the no-growth solution, and thus goes to extinction. Panel D is identical to Figure 5 of the main text.(TIF)

Figure S3Dynamical analysis. (A) 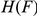 for the wild type at different IPTG concentrations: 0.1 mM (red), 1 mM (blue) and 100 mM (green). The steady-state solutions 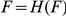 lie at the intersection of 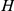 with the first bisecting line (black line); if it crosses it from above, the solution is stable (full dot), otherwise it is unstable (empty dot). (B) Time evolution of the growth rate of the wild type in 1 mM IPTG obtained by a discrete process (Equation 8 of Text S1, with a time step 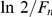 between step 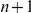 and 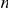; dots) and a continuous-time description (Equation 10 of Text S1; lines), for various initial growth rates. The generation time in minutes is 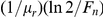, with 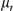 the growth rate of the reference strain measured to be 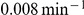. The full black line shows the stable steady state, the dashed line the unstable one. See Text S1 for definitions.(TIF)

Figure S4Fitness cost 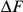 as a function (A) of the protein synthesis rate 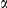, (B) of the protein concentration 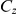. The dots show the measured fitness cost for different strains, in absence of IPTG (blue circles) and in 1 mM IPTG (mauve squares). The red dot shows the fitness cost measured for 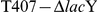 in 1 mM IPTG. The data shown in panel B are the same as those shown in Figure 2 of the main text. Error bars represent the standard error of the mean. The lines are the theoretical prediction, in absence of IPTG (blue) and in 1 mM IPTG (mauve). The dashed lines show the unstable solutions. The gray lines show the correlation of 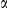 and 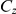 with 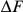 due to growth effects (see Text S1), for different values of 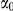. Starting from an initial selection coefficient (e.g., upon a change of medium), a given strain moves along a gray line toward the stable steady-state solution, and away from the unstable one.(TIF)

Figure S5Comparison of protein expression *(left)* and fitness cost 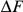 *(right)* on control strains. BW30270 is the wild type strain, T45 is a direct Datsenko-Wanner wild type construction and T273 is a transduction wild-type construction which went through the same procedures as all the *lac* operon mutants. Measurements were made in glycerol minimal medium without IPTG (white) and with 1 mM of IPTG (blue). Fitness was measured in competition against 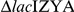. See Materials and Methods of the main text for a description of the strain constructions and competition experiments. The error bars represent the standard error of the mean.(TIF)

Figure S6 Expression levels of the different *lac* operator mutants. Protein expression was measured as described in Materials and Methods of the main text without IPTG (white) and with 1 mM of IPTG (blue). The error bars represent the standard error of the mean, with at least three replicates in each condition.
(TIF)

Figure S7Growth rate, in 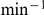, measured in the same conditions as described for the competition experiments, except each strain was grown separately. Every hour, for 10 hours, 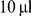 of the culture was taken and diluted appropriately, then plated on LB plates. Their mean lag phase was about 2 hours, therefore points 0, 1 hour and 2 hours were not used to estimate the growth rate. The growth rate 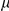 was estimated as the slope of the regression of 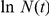 on time 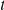, where 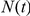 is the population size, such that: 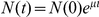. The error bars represent the standard error of the mean of 3 independent replicates. The Malthusian fitness 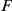 defined in Materials and Methods of the main text is equal to 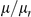, with the growth rate of the reference strain 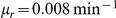.(TIF)

Figure S8Growth effects on gene expression and cell volume. (A) The protein concentration 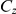 of a constitutively expressed gene has been proposed to correlate linearly with the growth rate 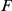 (red line), instead of the hyperbolic dependence dilution alone would induce (black line) [14]. (B) The cell volume 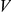 also correlates with 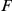; dots show experimental data taken from [13]; we choose to represent this correlation via a simple proportional dependence (red line). (C) Both correlations lead to a dependence 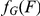 of the rate of protein synthesis on the growth rate 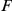 (red line; see Materials and Methods of the main text). Following [13], [14], the dependences are shown relative to the values at a growth rate 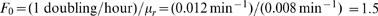. The highlighted area 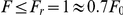 shows the range of growth rates relevant in this study.(TIF)

Figure S9Estimated maximal rate of expression at 1 doubling/hour 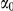 and ratio of repressed to unrepressed rates 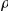 (see Materials and Methods of the main text), for all mutants used in this study. The wild type (red) and 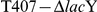 (orange) are barely distinguishable, as expected. In purple, the mutants which have a 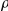 value very close to that of the wild type and are shown on Figure 5 of the main text (these are strains T319, T320, T378 and T379). In yellow, the whole operator mutants (T274, T275, T318). In green, the strain 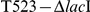. The values of 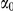 and 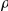 are reported in Table S1. Errors were computed as described in Text S1.(TIF)

Figure S10Statistical score of the model for a range of coefficients 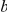 and 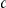, with 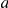 fixed at its fitted value. The higher the score, the lighter the shading color. The contours are drawn at scores −420, −450, −500, −550, −600, −650, −700, and −750. The highest score −417 is obtained for 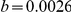 and 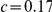 (red dot), significantly better than the best model with 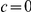 (which has score −426).(TIF)

Figure S11(A) Fitness cost 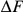 as a function of 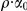, in absence of IPTG. (B) Fitness cost 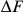 as a function of 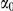, in 1 mM IPTG. Dots show the selection coefficient measured for different strains (error bars represent the standard error of the mean), lines are model predictions. In presence of IPTG, the stable solution shown as a full line in panel B was used to compute the score 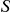 and fit the data. 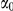 and 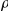 are estimated for each strain as explained in Materials and Methods of the main text. Errors were computed as described in Text S1.(TIF)

Table S1List of the strain studied, their *lac* O1 alleles and sequences (starting at the +1 site; underlined: mutations with respect to the wild type). The estimated values for the maximum rate of protein synthesis at 1 doubling/hour 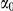 and ratio of repressed to unrepressed rates 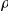 are also shown (see Materials and Methods of the main text). Errors were computed as described in Text S1.(PDF)

Table S2 List of the strains used in this study, their genotype and the way they were constructed.
(PDF)

Table S3 List of the plasmids used in this study, their relevant traits and the way they were obtained.
(PDF)

Table S4 List of the oligonucleotides used in this study, their sequence and the strain for the construction of which they were used.
(PDF)

Text S1 Supplementary Material.
(PDF)
