# Supplementary figures and images for: Nonlinear Fitness Landscape of a Molecular Pathway
Source: PLoS Genet. 2011 Jul 21;7(7):e1002160. doi: 10.1371/journal.pgen.1002160 (PMC3140986; doi:10.1371/journal.pgen.1002160)

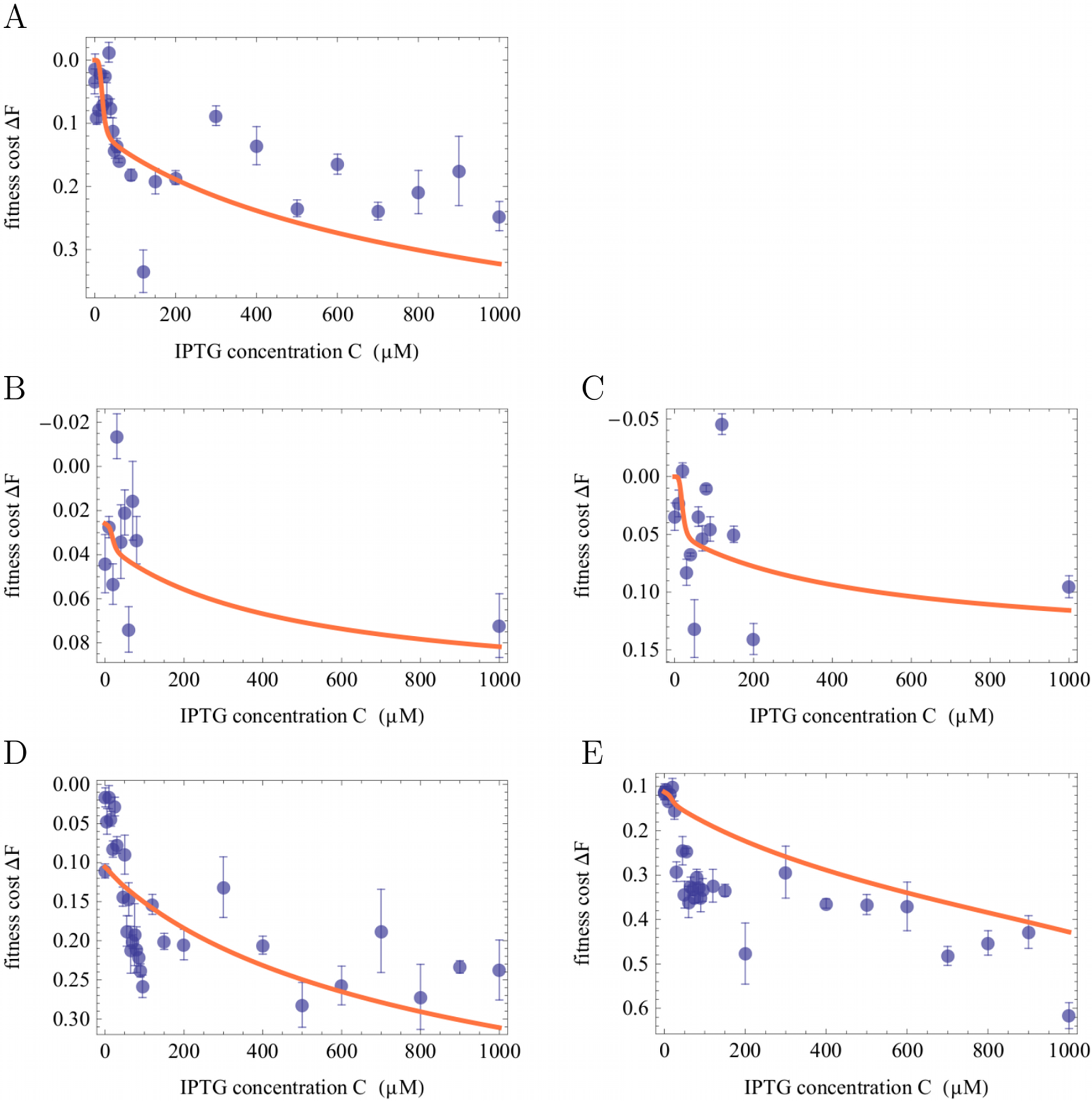

Supplement: Figure S1 — Fitness cost as a function of the IPTG concentration for five strains: (A) the wild type, (B) T274, (C) T320, (D) , (E) T275. The full lines are model predictions; dots show the experimental data (the error bars represent the standard error of the mean on 4 replicates, on 12 replicates for data at 0 and 1 mM IPTG). See Table S1 for a list of strains and the corresponding values of and . (TIF) [file pgen.1002160.s001.tif]

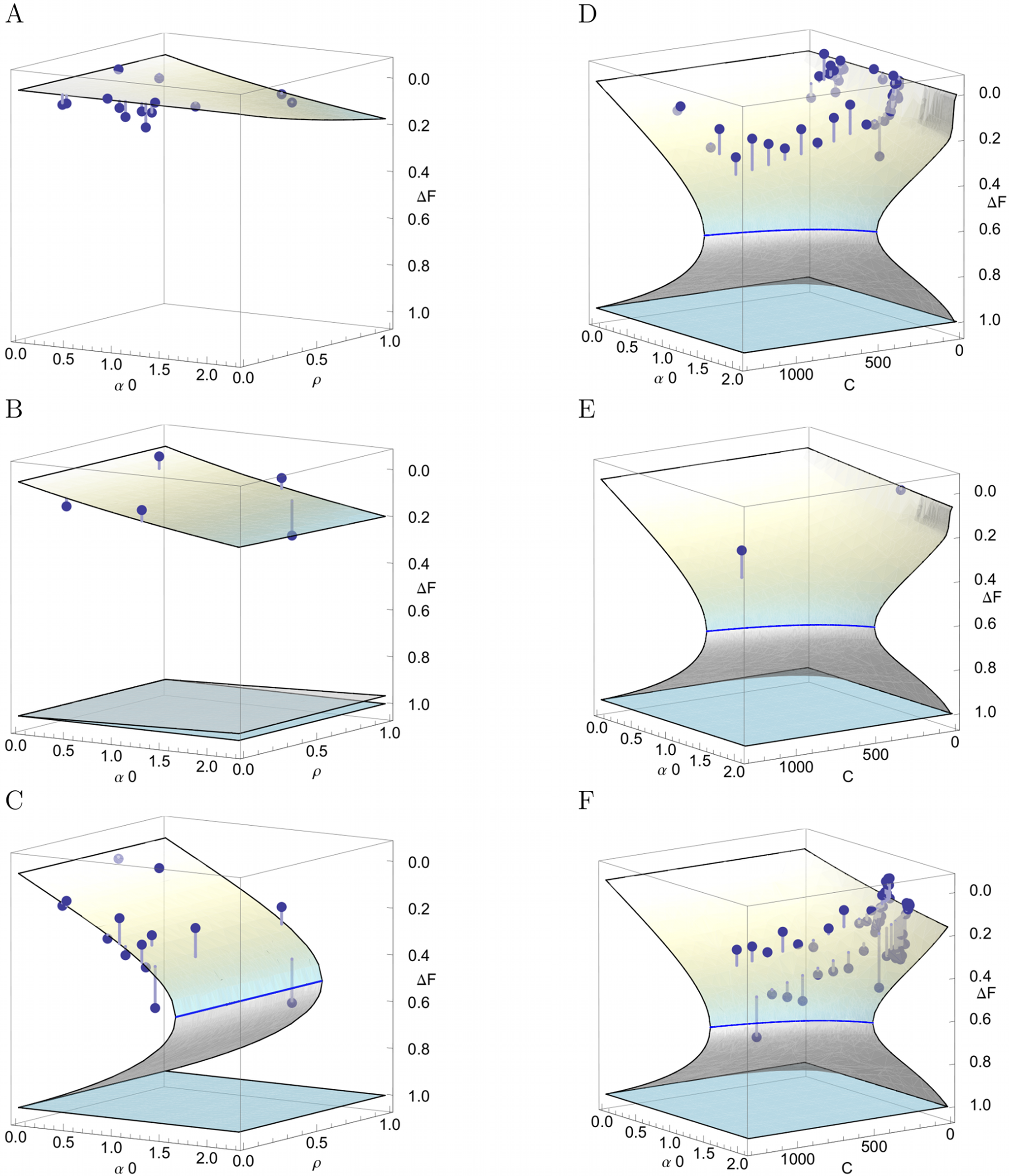

Supplement: Figure S2 — Fitness cost as a function of and , at fixed external IPTG concentration (A) , (B) and (C) ; as a function of and , at fixed (D) , (E) and (F) . The dots are the experimental data, the grey vertical bars show the distance between data and model prediction. Strains shown: (A) and (C) all operator mutants, wild type, (D) wild type, T319, T320, T378, T379; (B) wild type, T274, T275, T320, ; (E) T323; (F) T275, . The light-green surfaces show the stable solutions, the dark gray the unstable one. The blue line marks their boundary: when or the external IPTG concentration is increased beyond this “cliff”, the population falls on the no-growth solution, and thus goes to extinction. Panel D is identical to Figure 5 of the main text. (TIF) [file pgen.1002160.s002.tif]

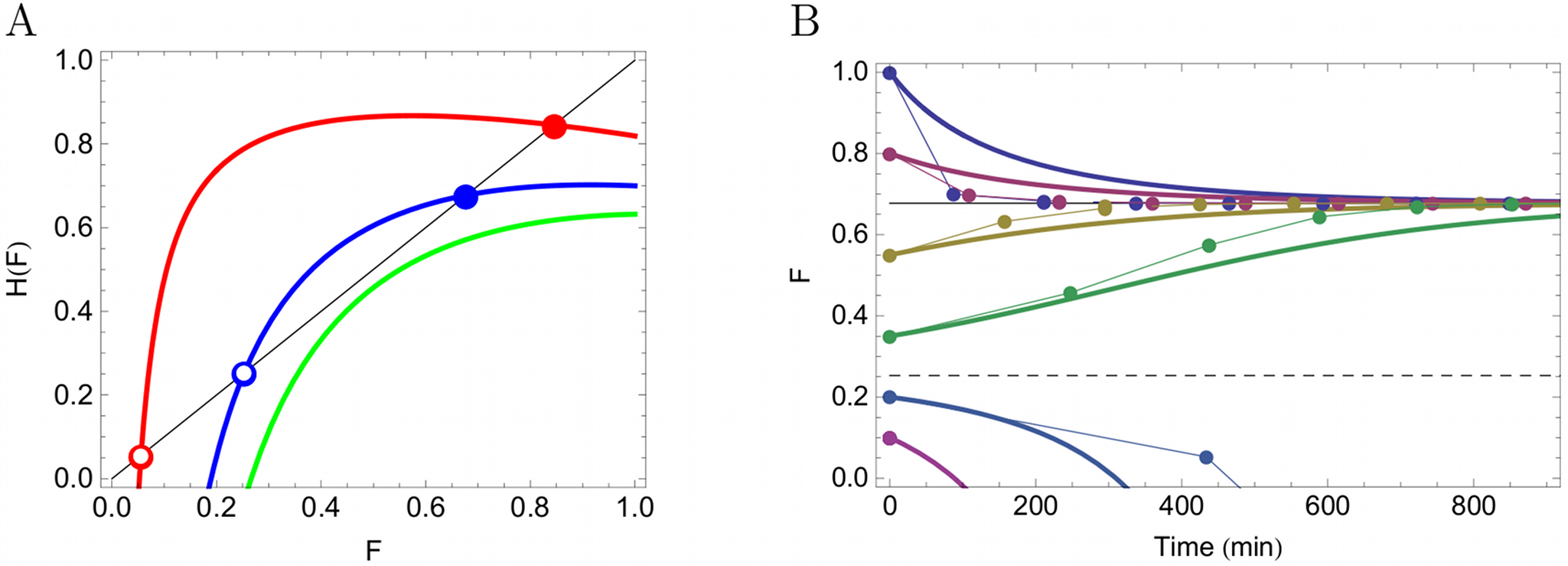

Supplement: Figure S3 — Dynamical analysis. (A) for the wild type at different IPTG concentrations: 0.1 mM (red), 1 mM (blue) and 100 mM (green). The steady-state solutions lie at the intersection of with the first bisecting line (black line); if it crosses it from above, the solution is stable (full dot), otherwise it is unstable (empty dot). (B) Time evolution of the growth rate of the wild type in 1 mM IPTG obtained by a discrete process (Equation 8 of Text S1, with a time step between step and ; dots) and a continuous-time description (Equation 10 of Text S1; lines), for various initial growth rates. The generation time in minutes is , with the growth rate of the reference strain measured to be . The full black line shows the stable steady state, the dashed line the unstable one. See Text S1 for definitions. (TIF) [file pgen.1002160.s003.tif]

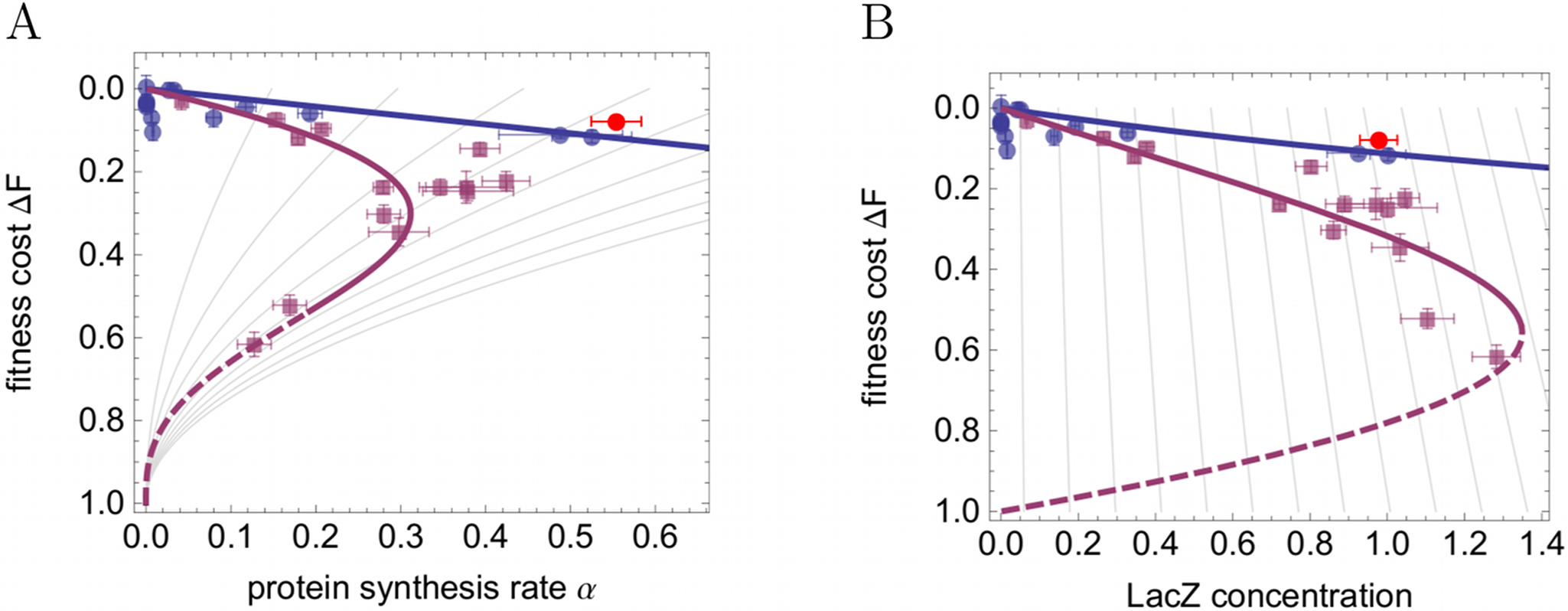

Supplement: Figure S4 — Fitness cost as a function (A) of the protein synthesis rate , (B) of the protein concentration . The dots show the measured fitness cost for different strains, in absence of IPTG (blue circles) and in 1 mM IPTG (mauve squares). The red dot shows the fitness cost measured for in 1 mM IPTG. The data shown in panel B are the same as those shown in Figure 2 of the main text. Error bars represent the standard error of the mean. The lines are the theoretical prediction, in absence of IPTG (blue) and in 1 mM IPTG (mauve). The dashed lines show the unstable solutions. The gray lines show the correlation of and with due to growth effects (see Text S1), for different values of . Starting from an initial selection coefficient (e.g., upon a change of medium), a given strain moves along a gray line toward the stable steady-state solution, and away from the unstable one. (TIF) [file pgen.1002160.s004.tif]

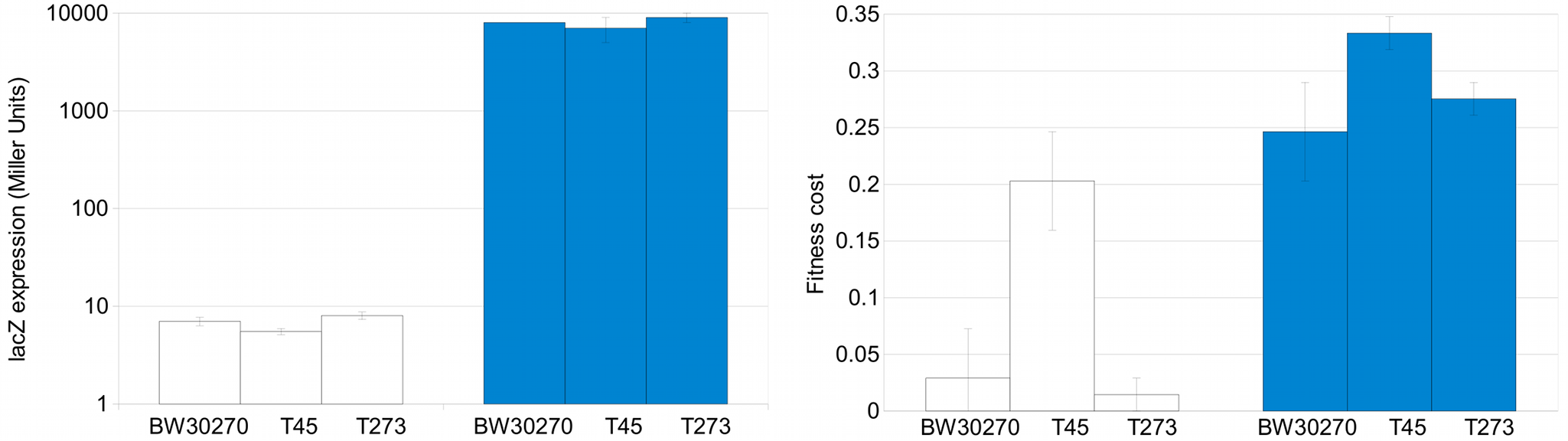

Supplement: Figure S5 — Comparison of protein expression (left) and fitness cost (right) on control strains. BW30270 is the wild type strain, T45 is a direct Datsenko-Wanner wild type construction and T273 is a transduction wild-type construction which went through the same procedures as all the lac operon mutants. Measurements were made in glycerol minimal medium without IPTG (white) and with 1 mM of IPTG (blue). Fitness was measured in competition against . See Materials and Methods of the main text for a description of the strain constructions and competition experiments. The error bars represent the standard error of the mean. (TIF) [file pgen.1002160.s005.tif]

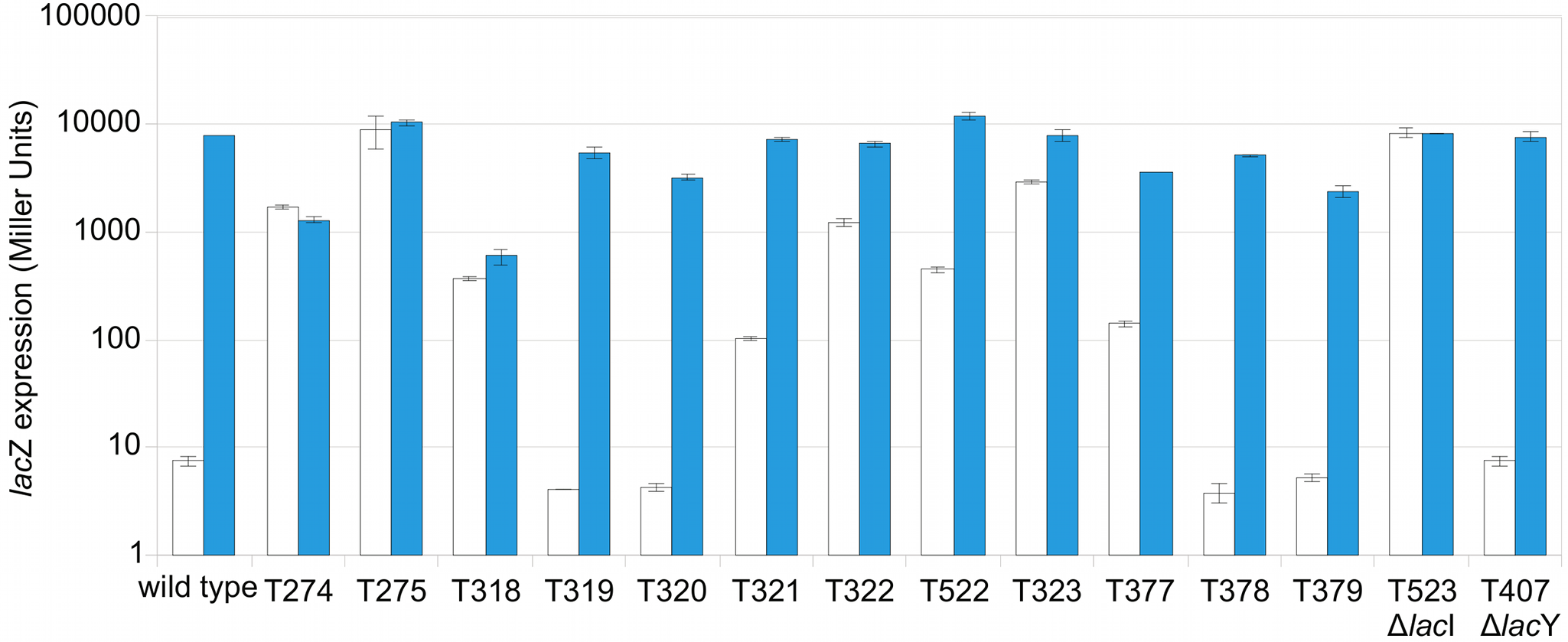

Supplement: Figure S6 — Expression levels of the different lac operator mutants. Protein expression was measured as described in Materials and Methods of the main text without IPTG (white) and with 1 mM of IPTG (blue). The error bars represent the standard error of the mean, with at least three replicates in each condition. (TIF) [file pgen.1002160.s006.tif]

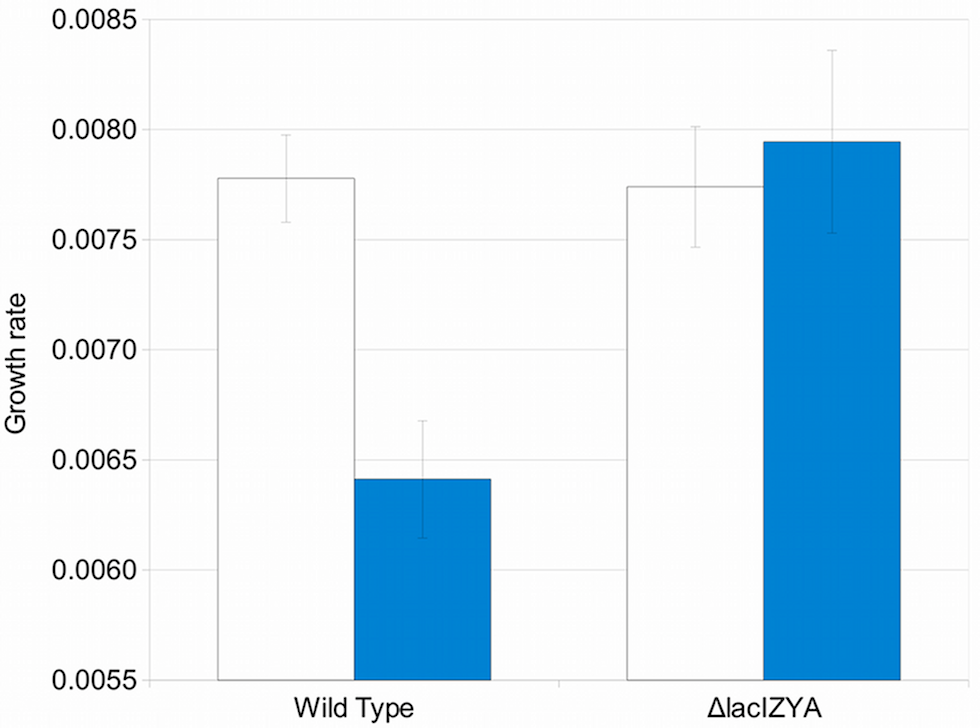

Supplement: Figure S7 — Growth rate, in , measured in the same conditions as described for the competition experiments, except each strain was grown separately. Every hour, for 10 hours, of the culture was taken and diluted appropriately, then plated on LB plates. Their mean lag phase was about 2 hours, therefore points 0, 1 hour and 2 hours were not used to estimate the growth rate. The growth rate was estimated as the slope of the regression of on time , where is the population size, such that: . The error bars represent the standard error of the mean of 3 independent replicates. The Malthusian fitness defined in Materials and Methods of the main text is equal to , with the growth rate of the reference strain . (TIF) [file pgen.1002160.s007.tif]

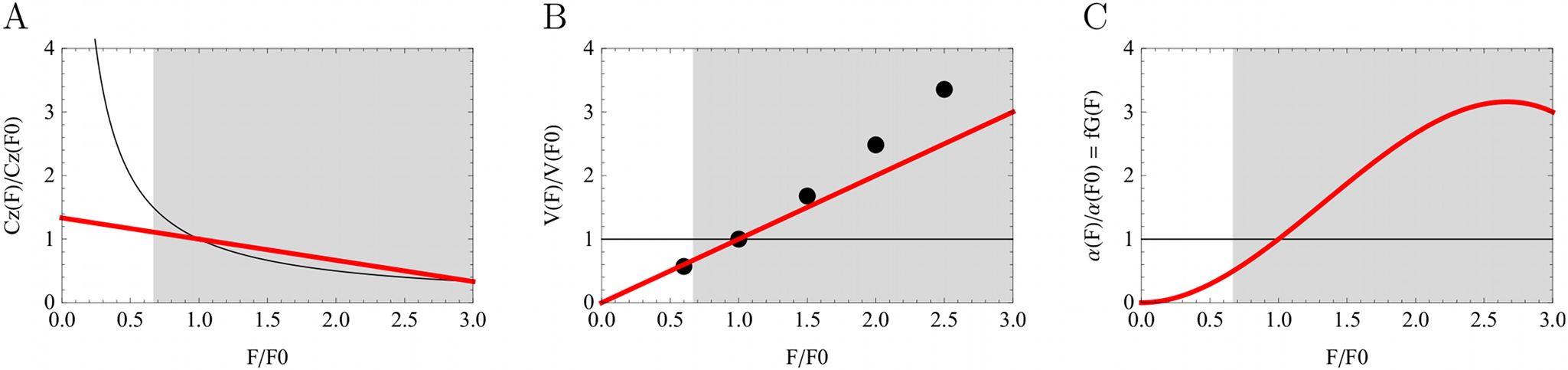

Supplement: Figure S8 — Growth effects on gene expression and cell volume. (A) The protein concentration of a constitutively expressed gene has been proposed to correlate linearly with the growth rate (red line), instead of the hyperbolic dependence dilution alone would induce (black line) [14]. (B) The cell volume also correlates with ; dots show experimental data taken from [13]; we choose to represent this correlation via a simple proportional dependence (red line). (C) Both correlations lead to a dependence of the rate of protein synthesis on the growth rate (red line; see Materials and Methods of the main text). Following [13], [14], the dependences are shown relative to the values at a growth rate . The highlighted area shows the range of growth rates relevant in this study. (TIF) [file pgen.1002160.s008.tif]

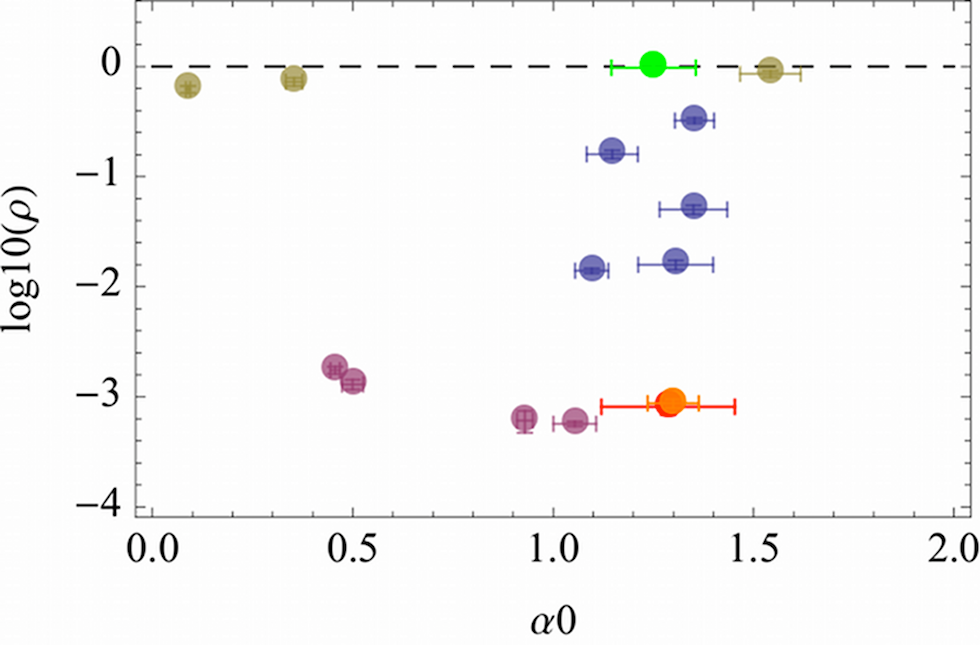

Supplement: Figure S9 — Estimated maximal rate of expression at 1 doubling/hour and ratio of repressed to unrepressed rates (see Materials and Methods of the main text), for all mutants used in this study. The wild type (red) and (orange) are barely distinguishable, as expected. In purple, the mutants which have a value very close to that of the wild type and are shown on Figure 5 of the main text (these are strains T319, T320, T378 and T379). In yellow, the whole operator mutants (T274, T275, T318). In green, the strain . The values of and are reported in Table S1. Errors were computed as described in Text S1. (TIF) [file pgen.1002160.s009.tif]

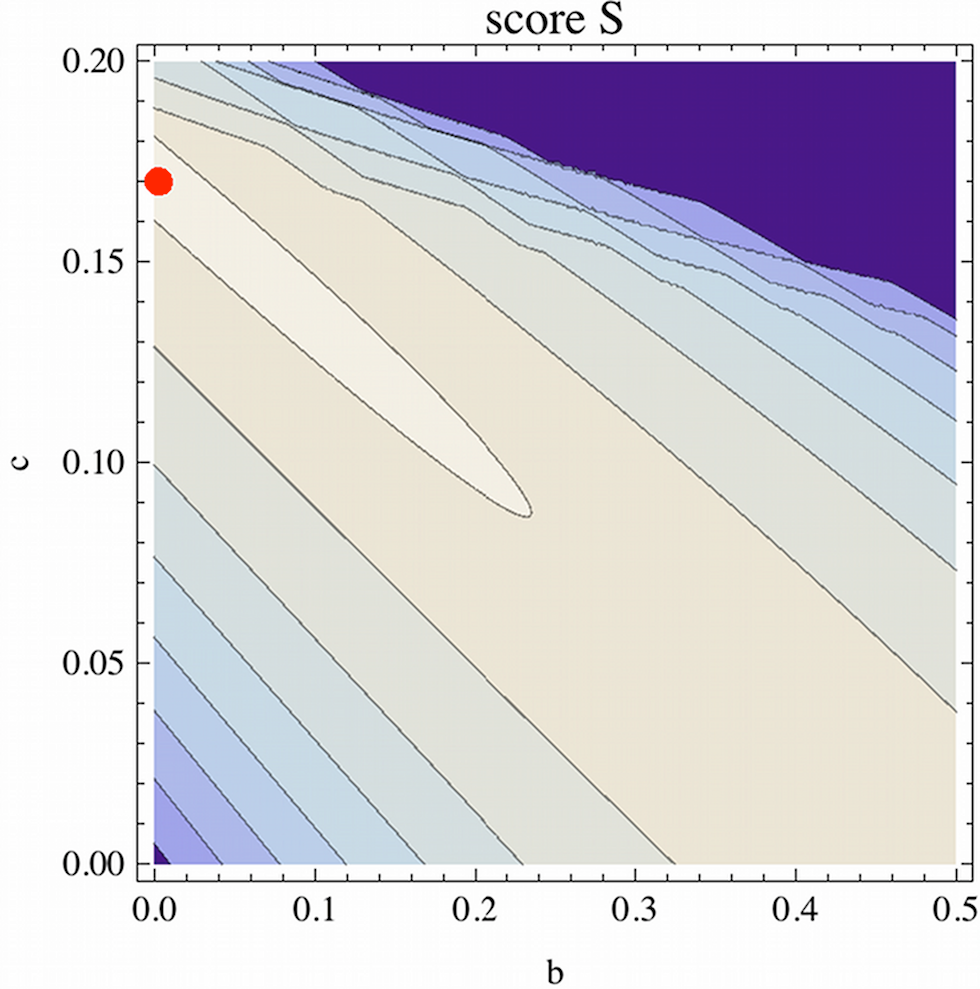

Supplement: Figure S10 — Statistical score of the model for a range of coefficients and , with fixed at its fitted value. The higher the score, the lighter the shading color. The contours are drawn at scores −420, −450, −500, −550, −600, −650, −700, and −750. The highest score −417 is obtained for and (red dot), significantly better than the best model with (which has score −426). (TIF) [file pgen.1002160.s010.tif]

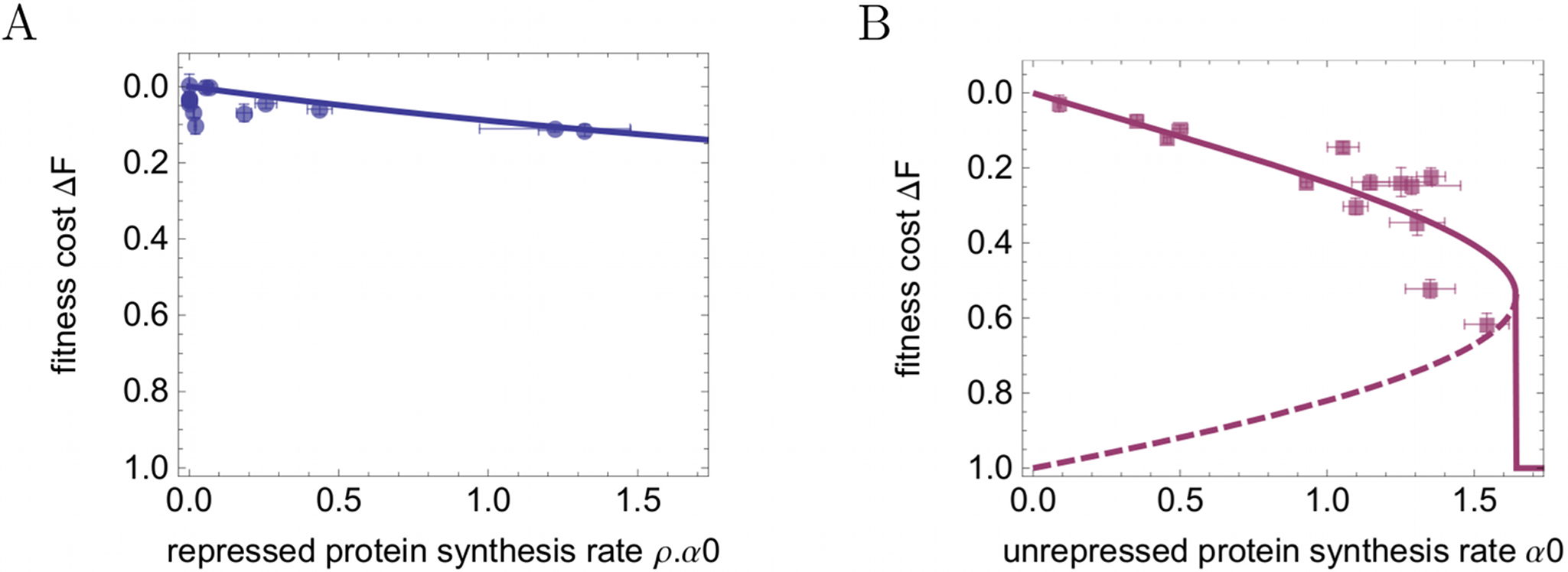

Supplement: Figure S11 — (A) Fitness cost as a function of , in absence of IPTG. (B) Fitness cost as a function of , in 1 mM IPTG. Dots show the selection coefficient measured for different strains (error bars represent the standard error of the mean), lines are model predictions. In presence of IPTG, the stable solution shown as a full line in panel B was used to compute the score and fit the data. and are estimated for each strain as explained in Materials and Methods of the main text. Errors were computed as described in Text S1. (TIF) [file pgen.1002160.s011.tif]
