# Supplementary material for: Nonlinear Fitness Landscape of a Molecular Pathway
Source: PLoS Genet. 2011 Jul 21;7(7):e1002160. doi: 10.1371/journal.pgen.1002160 (PMC3140986; doi:10.1371/journal.pgen.1002160)
Supplement: Table S1 — List of the strain studied, their lac O1 alleles and sequences (starting at the +1 site; underlined: mutations with respect to the wild type). The estimated values for the maximum rate of protein synthesis at 1 doubling/hour and ratio of repressed to unrepressed rates are also shown (see Materials and Methods of the main text). Errors were computed as described in Text S1. (PDF) [file pgen.1002160.s012.pdf]

strain	*lac*O1 allele / gene deleted	*lac*O1 sequence	α_0	ρ
BW30270	*lac*O1 (wild type)	AATTGTGAGCGGATAACAATT	1.29 ± 0.17	$(8.1 \pm 1.3)\,10^{-4}$
T274	*lac*O1-20R	ATCGCGACTGTCCACTGTGCA	0.35 ± 0.02	0.73 ± 0.06
T275	*lac*O1-20GCW	AGTGTCATTATACATCGATAG	1.54 ± 0.08	0.86 ± 0.06
T318	*lac*O1-20GCI	AATGCCACAGTCGCTCACCGG	0.089 ± 0.005	0.62 ± 0.04
T319	*lac*O1-SN2	ATTTGTGAGCGGATAACAATT	1.05 ± 0.05	$(5.7 \pm 0.3)\,10^{-4}$
T320	*lac*O1-SN3	AACTGTGAGCGGATAACAATT	0.50 ± 0.03	$(1.3 \pm 0.1)\,10^{-3}$
T321	*lac*O1-SN4	AATGGTGAGCGGATAACAATT	1.10 ± 0.04	$(1.4 \pm 0.07)\,10^{-2}$
T322	*lac*O1-SN5	AATTCTGAGCGGATAACAATT	1.15 ± 0.06	0.16 ± 0.01
T323	*lac*O1-SN8	AATTGTGCGCGGATAACAATT	1.35 ± 0.05	0.32 ± 0.02
T377	*lac*O1-SN9	AATTGTGATCGGATAACAATT	1.31 ± 0.09	$(1.6 \pm 0.1)\,10^{-2}$
T378	*lac*O1-SN12	AATTGTGAGCGCATAACAATT	0.93 ± 0.02	$(6.1 \pm 1.3)\,10^{-4}$
T379	*lac*O1-SN19	AATTGTGAGCGGATAACAGTT	0.46 ± 0.01	$(1.7 \pm 0.1)\,10^{-3}$
T522	*lac*O1-SN7	AATTGTAAGCGGATAACAATT	1.35 ± 0.08	$(5.0 \pm 0.5)\,10^{-2}$
T407-Δ*lac*Y	*lac*O1 (wild type) / *lac*Y deleted	AATTGTGAGCGGATAACAATT	1.30 ± 0.06	$(8.7 \pm 0.8)\,10^{-4}$
T523-Δ*lac*I	*lac*O1 (wild type) / *lac*I deleted	AATTGTGAGCGGATAACAATT	1.25 ± 0.10	0.98 ± 0.1
