# Supplementary material for: Nonlinear Fitness Landscape of a Molecular Pathway
Source: PLoS Genet. 2011 Jul 21;7(7):e1002160. doi: 10.1371/journal.pgen.1002160 (PMC3140986; doi:10.1371/journal.pgen.1002160)
Supplement: Table S2 — List of the strains used in this study, their genotype and the way they were constructed. (PDF) [file pgen.1002160.s013.pdf]

Strain	Genotype	Construction/Reference
BW30270	K12 MG1655 *rph*$^+$	*E. coli* genetic stock center
S4078	BW30270 Δ*lac*IZYA::pKD3-cm	BW30270/pKD46 x pKD3 PCR S911/S937
S4085	BW30270 Δ*lac*IZYA:: FRT	S4078 x pCP20 flp
T386	BW30270 Δ*lac*Y::pKD4-Kan	BW30270/pKD46 x pKD4 PCR T280/T281
T407	BW30270 Δ*lac*Y::FRT	T386 x pCP20 flp
T521	BW30270 Δ*lac*I::pKD4-Kan	BW30270/pKD46 x pKD4 PCR T361/T362
T523	BW30270 Δ*lac*I::FRT	T521 x pCP20 flp
S3974	BW30270 *ilv*G$^+$	[1]
S4197	BW30270 *ilv*G$^+$ Δ*lac*Z	S3974 Δ*lac*Z-pFDY217
T765	BW30270 *ilv*G$^+$ Δ*lac*I::pKD4-Kan Δ*lac*Z	S4197/pKD46 x pKD4 PCR (T361/T362)
T792	BW30270 Δ*lac*I::pKD4-Kan Δ*lac*Z	T765 x T4GT7(T765)
T807	BW30270 Δ*lac*I Δ*lac*Z	T792 x pCP20 flp
S4146	BW30270 Δ*lac*PO::pKD3-cm	BW30270/pKD46 x pKD3 PCR T125/T126
T45	BW30270	S4146/pKD46 x pKELP01 PCR (T123/T124)
T109	BW30270 *lac*O1-20R	S4146/pKD46 x pKELP04 PCR (T123/T124)
T46	BW30270 *lac*O1-20GCW	S4146/pKD46 x pKELP05 PCR (T123/T124)
T47	BW30270 *lac*O1-20GCI	S4146/pKD46 x pKELP06 PCR (T123/T124)
T48	BW30270 *lac*O1-SN2	S4146/pKD46 x pKELP07 PCR (T123/T124)
T49	BW30270 *lac*O1-SN3	S4146/pKD46 x pKELP08 PCR (T123/T124)
T50	BW30270 *lac*O1-SN4	S4146/pKD46 x pKELP09 PCR (T123/T124)
T110	BW30270 *lac*O1-SN5	S4146/pKD46 x pKELP10 PCR (T123/T124)
T111	BW30270 *lac*O1-SN7	S4146/pKD46 x pKELP12 PCR (T123/T124)
T112	BW30270 *lac*O1-SN8	S4146/pKD46 x pKELP13 PCR (T123/T124)
T265	BW30270 *lac*O1-SN9	S4146/pKD46 x pKELP14 PCR (T123/T124)
T267	BW30270 *lac*O1-SN12	S4146/pKD46 x pKELP17 PCR (T123/T124)
T268	BW30270 *lac*O1-SN19	S4146/pKD46 x pKELP16 PCR (T123/T124)
T218	BW30270 Δ*lac*PO::pKD3-cm	BW30270 x T4GT7(S4146)
T273	BW30270	T218 x T4GT7(T108)
T274	BW30270 *lac*O1-20R	T218 x T4GT7(T109)
T275	BW30270 *lac*O1-20GCW	T218 x T4GT7(T46)
T318	BW30270 *lac*O1-20GCI	T218 x T4GT7(T47)
T319	BW30270 *lac*O1-SN2	T218 x T4GT7(T48)
T320	BW30270 *lac*O1-SN3	T218 x T4GT7(T49)
T321	BW30270 *lac*O1-SN4	T218 x T4GT7(T50)
T322	BW30270 *lac*O1-SN5	T218 x T4GT7(T110)
T522	BW30270 *lac*O1-SN7	T218 x T4GT7(T111)
T323	BW30270 *lac*O1-SN8	T218 x T4GT7(T112)
T377	BW30270 *lac*O1-SN9	T218 x T4GT7(T265)
T378	BW30270 *lac*O1-SN12	T218 x T4GT7(T267)
T379	BW30270 *lac*O1-SN19	T218 x T4GT7(T268)

[1] G Raja Venkatesh, Frant Carlot Kembou Koungni, Andreas Paukner, Thomas Stratmann, Birgit Blissenbach, and Karin Schnetz, "BglJ-RcsB heterodimers relieve repression of the *Escherichia coli bgl* operon by H-NS," J. Bacteriol. , JB.00807–10 (2010).
