# Supplementary material for: Nonlinear Fitness Landscape of a Molecular Pathway
Source: PLoS Genet. 2011 Jul 21;7(7):e1002160. doi: 10.1371/journal.pgen.1002160 (PMC3140986; doi:10.1371/journal.pgen.1002160)
Supplement: Table S3 — List of the plasmids used in this study, their relevant traits and the way they were obtained. (PDF) [file pgen.1002160.s014.pdf]

Plasmid	Structure	Construction or reference
pKD3	FRT-flanked cat gene in *oriR*γ replicon requiring the *pir* gene product	[1]
pKD4	FRT-flanked kan gene in *oriR*γ replicon requiring the *pir* gene product	[1]
pCP20	flp-recombinase repts catR ampR	[1]
pKEM72	pUV5-proV(+1 to+303)-rrnBT1 in pUC12 ampR	laboratory collection
pKELP01	wild type *lac* promoter (-90 to +163) - rrnBT1 in pUC12, ampR	MG1655 PCR T121/T122 in pKEM72 XbaI SalI
pKELP04	*lac* promoter *lac*O1-20R(-90 to +163) - rrnBT1 in pUC12, AmpR	pKELP01 PCR T131/S133 and T132/T24 in pKEM72 XbaI SalI
pKELP05	*lac* promoter *lac*O1-GCW(-90 to +163) - rrnBT1 in pUC12, AmpR	pKELP01 PCR T133/S133 and T134/T24 in pKEM72 XbaI SalI
pKELP06	*lac* promoter *lac*O1-GCI(-90 to +163) - rrnBT1 in pUC12, AmpR	pKELP01 PCR T135/S133 and T136/T24 in pKEM72 XbaI SalI
pKELP07	*lac* promoter *lac*O1-SN2(-90 to +163) - rrnBT1 in pUC12, AmpR	pKELP01 PCR T150/S133 and T151/T24 in pKEM72 XbaI SalI
pKELP08	*lac* promoter *lac*O1-SN3(-90 to +163) - rrnBT1 in pUC12, AmpR	pKELP01 PCR T152/S133 and T153/T24 in pKEM72 XbaI SalI
pKELP09	*lac* promoter *lac*O1-SN4(-90 to +163) - rrnBT1 in pUC12, AmpR	pKELP01 PCR T154/S133 and T155/T24 in pKEM72 XbaI SalI
pKELP10	*lac* promoter *lac*O1-SN5(-90 to +163) - rrnBT1 in pUC12, AmpR	pKELP01 PCR T156/S133 and T157/T24 in pKEM72 XbaI SalI
pKELP12	*lac* promoter *lac*O1-SN7(-90 to +163) - rrnBT1 in pUC12, AmpR	pKELP01 PCR T160/S133 and T161/T24 in pKEM72 XbaI SalI
pKELP13	*lac* promoter *lac*O1-SN8(-90 to +163) - rrnBT1 in pUC12, AmpR	pKELP01 PCR T162/S133 and T163/T24 in pKEM72 XbaI SalI
pKELP14	*lac* promoter *lac*O1-SN9(-90 to +163) - rrnBT1 in pUC12, AmpR	pKELP01 PCR T164/S133 and T165/T24 in pKEM72 XbaI SalI
pKELP16	*lac* promoter *lac*O1-SN19(-90 to +163) - rrnBT1 in pUC12, AmpR	pKELP01 PCR T184/S133 and T185/T24 in pKEM72 XbaI SalI
pKELP17	*lac* promoter *lac*O1-SN12(-90 to +163) - rrnBT1 in pUC12, AmpR	pKELP01 PCR T170/S133 and T171/T24 in pKEM72 XbaI SalI
pFDY217	*lac*I *lac*OP[Δ*lac*Z] *lac*Y *lac*A pSC101-repTS tetR	[2]

[1] K A Datsenko and B L Wanner, "One-step inactivation of chromosomal genes in *Escherichia coli* K-12 using PCR products," Proc Natl Acad Sci U S A **97**, 6640–6645 (2000).

[2] Sudhanshu Dole, Sandra Kühn, and Karin Schnetz, "Post-transcriptional enhancement of *Escherichia coli bgl* operon silencing by limitation of BglG-mediated antitermination at low transcription rates," Mol Microbiol **43**, 217–226 (2002).
