# Supplementary material for: Nonlinear Fitness Landscape of a Molecular Pathway
Source: PLoS Genet. 2011 Jul 21;7(7):e1002160. doi: 10.1371/journal.pgen.1002160 (PMC3140986; doi:10.1371/journal.pgen.1002160)
Supplement: Table S4 — List of the oligonucleotides used in this study, their sequence and the strain for the construction of which they were used. (PDF) [file pgen.1002160.s015.pdf]

Oligonucleotide	Sequence (5'-3')
S911	GTTCCTGCGCTTTGTTCATGCCGGATGCGGCTAATGTAGAGTGTAGGCT GGAGCTGCTTCG
S937	ATGATAGCGCCCGGAAGAGAGTCAATTCAGGGTGGTGAATCATATGAAT ATCCTCCTTAGTTCCTATTCC
T125	AGCGCAACGCAATTAATGTGAGTTAGCTCACTCATTAGGCACCGTGTAG GCTGGAGCTGCTTCG
T126	TCGCTATTACGCCAGCTGGCGAAAGGGGGATGTGCTGCAAGGCGATTA AGCATATGAATATCCTCCTTAGTTCCTATTCC
T200	CCGCATCATCTTCGGCATTTTTGCCCCATGCAAACGGGAAGTGGGAAT GGACCGTGTAGGCTGGAGCTGCTTCG
T201	TACTGTTTCTCCATACCCGTTTTTTTGGATGGAGTGAAACGATGGCGAT CATATGAATATCCTCCTTAGTTCCTATTCC
T280	AACCGGGCAGGCCATGTCTGCCCGTATTTCGCGTAAGGAAATCCATTC GTGTAGGCTGGAGCTGCTTCG
T281	TGATATGTTGGTCGGATAAGGCGCTCGCGCCGCATCCGACATTGATTG CCATATGAATATCCTCCTTAGTTCCTATTCC
T361	CGCAGGCTATTCTGGTGGCCGGAAGGCGAAGCGGCATGCATTTACGT TGACACCATCGTGTAGGCTGGAGCTGCTTCG
T362	TCGGGAAACCTGTCGTGCCAGCTGCATTAATGAATCGGCCAACGCGC GGGGAGACATATGAATATCCTCCTTAGTTCCTATTCC
S133	GTGGATAACCGTATTACCGC
T24	GAAAAGTGCCACCTGACGTCTAA
T121	GATAGTCGACCAGTGAGCGCAACGCAATTAATG
T122	GATATCTAGATCTTCGCTATTACGCCAGCTGG
T131	ATCGCGACTGTCCACTGTGCATCACACAGGAAACAGCTATGACC
T132	TGCACAGTGGACAGTCGCGATCCACACAACATACGAGCCGG
T133	TGTGGAGTGTCATTATACATCGATAGTCACACAGGAAACAGCTATGACC
T134	GTGACTATCGATGTATAATGACACTCCACACAACATACGAGCCGG
T135	AATGCCACAGTCGCTCACCGGTCACACAGGAAACAGCTATGACC
T136	CCGGTGAGCGACTGTGGCATTCCACACAACATACGAGCCGG
T150	ATTTGTGAGCGGATAACAATTTCACACAGGAAACAGCTATGACC
T151	AATTGTTATCCGCTCACAAATCCACACAACATACGAGCCGG
T152	AACTGTGAGCGGATAACAATTTCACACAGGAAACAGCTATGACC
T153	AATTGTTATCCGCTCACAGTTCCACACAACATACGAGCCGG
T154	AATGGTGAGCGGATAACAATTTCACACAGGAAACAGCTATGACC
T155	AATTGTTATCCGCTCACCATTCCACACAACATACGAGCCGG
T156	AATTCTGAGCGGATAACAATTTCACACAGGAAACAGCTATGACC
T157	AATTGTTATCCGCTCAGAATTCCACACAACATACGAGCCGG
T160	AATTGTAAGCGGATAACAATTTCACACAGGAAACAGCTATGACC
T161	AATTGTTATCCGCTTACAATTCCACACAACATACGAGCCGG
T162	AATTGTGCGCGGATAACAATTTCACACAGGAAACAGCTATGACC
T163	AATTGTTATCCGCGCACAATTCCACACAACATACGAGCCGG
T164	AATTGTGATCGGATAACAATTTCACACAGGAAACAGCTATGACC
T165	AATTGTTATCCGATCACAATTCCACACAACATACGAGCCGG
T170	AATTGTGAGCGCATAACAATTTCACACAGGAAACAGCTATGACC
T171	AATTGTTATGCGCTCACAATTCCACACAACATACGAGCCGG
T184	AATTGTGAGCGGATAACAGTTTCACACAGGAAACAGCTATGACC
T185	AACTGTTATCCGCTCACAATTCCACACAACATACGAGCCGG
