# Supplementary material for: Nonlinear Fitness Landscape of a Molecular Pathway
Source: PLoS Genet. 2011 Jul 21;7(7):e1002160. doi: 10.1371/journal.pgen.1002160 (PMC3140986; doi:10.1371/journal.pgen.1002160)
Supplement: Text S1 — Supplementary Material. (PDF) [file pgen.1002160.s016.pdf]

Nonlinear Fitness Landscape of a Molecular Pathway

Supplementary Material

Lilia Perfeito,[1,2,*] Stéphane Ghozzi,[2] Johannes Berg,[2] Karin Schnetz,[1] and Michael Lässig[2,†]

[1] *Institut für Genetik, Universität zu Köln, Zülpicherstrasse 47a, 50674 Cologne, Germany*
[2] *Institut für Theoretische Physik, Universität zu Köln, Zülpicherstrasse 77, 50937 Cologne, Germany*

(Dated: May 29, 2011)

* lperfeit@uni-koeln.de
† lassig@thp.uni-koeln.de

CONTENTS

I. Additional experiments and controls 2
A. Absolute growth rates and toxicity of IPTG 2
B. Depletion of IPTG 2
C. Fitness of *lac*O1 mutants before transduction 3
D. Strain, plasmid and oligonucleotide lists 3
E. Expression parameters 3

II. The modeling 3
A. Detailed transport model 3
B. Growth effects on gene expression and cell volume 4
C. Statistical score, model and data comparison 5
D. Dynamical analysis 6

III. Genotype-environment interactions 6

IV. Error propagation 7

References 7

I. ADDITIONAL EXPERIMENTS AND CONTROLS

A. Absolute growth rates and toxicity of IPTG

One of the possible sources of the additional cost in IPTG is the direct toxicity of this molecule. IPTG was previously proposed to be toxic, with *lac*A conferring protection against it [1]. It was also observed that an intermediate product of lactose metabolism might be toxic [2]. In order to test the toxicity of IPTG, we measure the absolute growth rate of the reference strain (which does not have any of the *lac* genes) and of the wild type at 0 and 1 mM IPTG (see Figure S7). The reference strain is still able to internalize IPTG through the cell membrane but, since it lacks LacY, the deleterious effect of the activity of this protein is absent. No effect of IPTG on the reference strain is observed, indicating that it is not toxic [1]. This measurement also confirms that the growth rate of the reference is the same at all IPTG concentrations used in this work.

The reference strain does not have *lac*Y and the internal concentration of IPTG ($C_{\rm int}$) it achieves is likely to be much lower than that of the wild type (see below). Is IPTG toxic at high internal concentrations? We can estimate the increase in $C_{\rm int}$ due to the presence of LacY from the differences in the IPTG induction curves of mutants with and without this protein because the expression rate of the *lac* promoter is a function of $C_{\rm int}$. Kuhlman and co-workers have shown that the maximum of induction was reached when the external IPTG concentration C was 30 μM for the wild type and 200 μM for a LacY$^-$ strain [4]. It appears thus that the presence of LacY causes a ~ 10 fold increase in $C_{\rm int}$. At steady state the internal and external concentrations of IPTG are identical for the reference strain ($C = C_{\rm int}$) because the flux of inducer through the membrane is expected to be proportional to concentration differences between the outside and inside of the cell [5, 6]. Thus the internal concentration of IPTG for the wild type growing at 100 μM of IPTG should be similar to the internal concentration of the reference growing at 1 mM. We observe a fitness cost for the wild type at $C = 100$ μM but no effect on the reference at $C = 1$ mM (see Figures S1 and S7). Furthermore, if toxicity was a major effect, the reference would be expected to be more sensitive than the wild type because it lacks *lac*A [1]. Hence we conclude that the supplementary cost associated with LacY in presence of IPTG cannot be entirely attributed to toxicity *per se*.

B. Depletion of IPTG

Since cells are internalizing IPTG, it is possible that they reduce its concentration in the medium. A depletion of external inducer would stretch the x-axis on Figure S1. If depletion is an important effect, the induction curve for the

[1] Dekel and Alon also did not measure any effect of 150 μM IPTG on the growth rate of a *lac* mutant [3].

wild type at low IPTG concentrations will deviate from the prediction based on the external concentration. As can be observed in Figure S1, the prediction matches well the experimental data, indicating that at low concentrations depletion is not significant. Furthermore, fitness reaches its lowest value at 200 μM and does not change further with increasing IPTG (Figure S1), showing that at high concentrations IPTG is in excess and any depletion that might exist does not affect the results. We conclude that depletion of IPTG from the medium is not a major factor during the competitions.

C. Fitness of *lac*O1 mutants before transduction

In order to control for any fitness effects caused by the strain construction method, a wild type strain is constructed through the same steps as the operator mutant strains. Strain T45 is constructed following [7] by replacing the wild type *lac* operon with a chloramphenicol resistance cassette and subsequently replacing this cassette with the wild type *lac* promoter again (see Table S2 for details). This strain is then competed against the reference strain (Δ*lac*IZYA) and its protein expression is measured. The procedures used are the same as described in Materials and Methods of the main text. As can be seen in Figure S5, the constructed strain has a similar protein expression but a clear decrease in fitness to the "real" wild type strain (BW30270). We were able to eliminate this effect by reconstructing the strains with T4GT7-mediated transduction (see Materials and Methods of the main text): the new wild type built in this way (T273) cannot be differentiated from BW30270, see Figure S5. Throughout this work, "wild type" refers to the BW30270 strain.

D. Strain, plasmid and oligonucleotide lists

The strains, oligonucleotides, *lac*O1 operator sequences and plasmids used in this work are shown in Tables S1, S2, S3 and S4.

E. Expression parameters

Figure S6 shows the expression levels measured for all strains. Their estimated maximal rate of expression at 1 doubling/hour α_0 and ratio of repressed to unrepressed rates ρ are shown in Figure S9 (see Materials and Methods of the main text). The corresponding values are listed in Table S1. α_0 and ρ depend only on the sequence of the *lac*O1 operator and, as Figure S9 shows, all operator mutants are distinguishable using these variables, making them ideal genetic variables.

As expected: (i) The strain T523-Δ*lac*I as a ρ value equal to 1, and an α_0 value close to that of the wild type (green and red dots respectively in Figure S9). (ii)T407-Δ*lac*Y has α_0 and ρ values identical to those of the wild type (orange and red dots respectively). (iii) Finally, mutants with the whole operator sequence mutated have ρ close to 1, i.e. very poorly bind the LacI repressor (yellow dots).

Also, the different operator mutants cover a wide region of α_0 and ρ values, thus allowing us to check our model in a large part of the genotypic space.

Let us note here that *not considering* growth effects on gene expression leads to an unrealistic $\rho = 1.8$ for the T275 mutant (implying higher expression in absence of inducer). This is a hint that such effects apply here; further model comparisons are discussed in Section II C below.

II. THE MODELING

A. Detailed transport model

The model presented in the main text does not take into account diffusion through the cell membrane or efflux through LacY. Here we model IPTG transport in more detail and show that Equation 1 of the main text is a good approximation and retains the relevant growth dependences.

The first step in IPTG uptake is diffusion through the outer membrane into the periplasmic space (see [8] and references therein). We make the simplest assumption that this first step is fast and that IPTG can diffuse perfectly to inner membrane (where the LacY molecules are located). We denote by $\tilde{\gamma}$ the absolute (not normalized) net

transport rate per LacY molecule. Taking efflux into account, it can be written [9]:

$$\tilde{\gamma} \;=\; E_y \frac{C - C_{\text{int}}(K_p/K_e)}{K_p + C}, \tag{1}$$

where E_y is the maximum transport rate per LacY molecule, K_p and K_e are the half saturation constants for influx and efflux respectively, and C and C_{int} are the external and internal concentrations of IPTG respectively.

Values for LacY kinetic parameters have been reported, but they vary greatly between different studies and depend on the LacY substrate, the strain, the temperature, and the culture medium [5, 6, 10]. To our knowledge, they were not measured for IPTG. However, K_e is consistently two orders of magnitude larger than K_p. Furthermore, as explained Section I A above, C_{int} is expected to be less than 100 higher than C (possibly only about 10 fold higher). Hence we expect $C_{\text{int}}(K_p/K_e)$ to be small compared to C and we can neglect efflux in the equation above: $\tilde{\gamma} \approx E_y C/(K_p + C)$. The normalized transport rate γ is defined as $\tilde{\gamma}(C)/\tilde{\gamma}(C_1)$, where $C_1 = 1$ mM. This leads to the expression of γ written in Materials and Methods of the main text:

$$\gamma \;\approx\; \frac{C}{C_1}\frac{K_p + C_1}{K_p + C}. \tag{2}$$

The cost related to toxicity of IPTG or another transported molecule (the third term in Equation 1 of the main text) is proportional to its internal concentration C_{int}. To estimate C_{int} we consider the fluxes into the cell. The first influx is due to transport through LacY, and equals $C_y\,\tilde{\gamma}$, with C_y the concentration of LacY molecules. Secondly, there can be diffusion through the cell membrane. The resulting flux is proportional to the difference between the external and internal concentrations, and, denoting by k_d the diffusion constant, is equal to $k_d(C - C_{\text{int}})$. Lastly, molecules are lost by cell division, the pace of which is the growth rate measured in min^{-1} and denoted by μ. This results in a flux $-\mu\, C_{\text{int}}$. At steady state, all three fluxes cancel each other:

$$C_y\,\tilde{\gamma} + k_d(C - C_{\text{int}}) - \mu\, C_{\text{int}} = 0. \tag{3}$$

Solving this equation for the internal concentration leads to

$$C_{\text{int}} \;=\; \frac{k_d\, C}{k_d + \mu} + \frac{C_y\,\tilde{\gamma}}{k_d + \mu}. \tag{4}$$

The first term of this equation shows the accumulation due to diffusion, and is of same amplitude for the studied and reference strains. However, since no growth reduction was measured for the reference strain in IPTG, and lactose or protons do not diffuse (*i.e.*, they have small k_d), the contribution of this term can be neglected in all cases, and $C_{\text{int}} \approx C_y\tilde{\gamma}/(k_d + \mu)$.

We now express the cost related to toxicity in terms of the phenotype $\Gamma = N_y\,\gamma$, with N_y the number of LacY molecules, and the cell volume V. The concentration of LacY is equal to its number divided by the volume, such that Γ is proportional to $VC_y\tilde{\gamma}$. Lactose and protons diffuse very little, and k_d is negligible compared to μ. Thus, the internal concentration of these molecules is approximately $C_y\tilde{\gamma}/\mu$. With μ being proportional to F, this leads to a toxicity proportional to Γ/FV, as stated in the main text.

On the other hand, IPTG diffuses well through the cell membrane, and its diffusion constant k_d is much larger than the typical growth rate in our experiments (close to 0.008 min^{-1}, whereas k_d has been measured to be 0.1 min^{-1} for TMG [9], which is known to diffuse much less than IPTG). A toxicity of IPTG would thus contribute a term proportional to Γ/V to the fitness cost. Considering a supplemental term in Γ/V would add one coefficient to be fitted, without qualitatively changing our results. Moreover, the accumulation of protons is more likely to be the direct cause of the fitness cost because: (i) IPTG it is not likely to be toxic (see above); (ii) it was shown that cellular pH is disrupted by the presence of inducer in the medium [11]; (iii) it was suggested that osmotic effects of IPTG do not contribute to the overall fitness cost in an important way [11]. This is in agreement with our results, which suggest a major contribution from the term in Γ/FV.

B. Growth effects on gene expression and cell volume

Correlations between cellular physiology, growth rate and gene expression have been recently highlighted [12, 13], leading us to the introduction of the factor f_G (see Material and Methods of the main text). Furthermore, the protein concentration C_z would unrealistically tend to infinity as the growth rate F tends to zero if C_z correlated to growth through dilution only (implying $C_z \sim 1/F$). For simplicity, we follow [13] and consider a linear correlation between the LacZ concentration C_z and the growth rate F at all values of F (as illustrated in Figure S8A). Such correlations

were observed for constitutively expressed genes when cells were grown in media of different nutrient qualities. It is thus not obvious *a priori* that they should apply here (for instance, an inverse correlation is observed when cells are grown in a translation inhibiting drug [13]).

As explained in the Materials and Methods of the main text, the protein production rate α can be estimated from C_z by considering that, at steady state $\alpha = C_z V F$, with V the cell volume. In order to understand how the correlation between C_z and F extends to α, we have to take into account that V also correlates with the growth rate F. We choose a linear function for $V(F)$, shown in Figure S8B (red line) along with previous estimates (black dots) [12]. An exponential dependence for $V(F)$ leads to a more realistic finite volume as the growth rate goes to zero and was observed for the bacterium *Salmonella typhimurium* [14]. However, both dependences agree equally well with the data up to $1.5\,F_0$, and lead to similar results. We present results with the simplest dependence $V \sim F$.

Once the volume dependence on the growth rate F is taken into account, the rate of protein synthesis α of a constitutively expressed gene also has to correlate with F to produce the linear dependence of protein concentration. We denote by f_G this dependence (see Materials and Methods of the main text for the full expression and derivation) and plot it in Figure S8C. As discussed in Sections I E above and II C below, the inclusion of f_G helps explain our data.

C. Statistical score, model and data comparison

In order to measure the agreement between data and theory, we compute a statistical score S. By maximizing it, we can find the best model parameters and measure the goodness of the fit.

We consider that the growth rate F and the phenotypes α and Γ depend on three fundamental, independent variables: two fixed by *lac*O1 sequence (α_0 and ρ), one by the environment (the external IPTG concentration C). Moreover the cell volume V and the protein synthesis rate α depend on F. Applying these dependences to Equation 1 of the main text, decomposing Γ in $\alpha\gamma/F$ in their dependences on the independent variables (see Materials and Methods of the main text) and solving for F produces:

$$F \;=\; F_r - a \times \alpha(\alpha_0, \rho, C, F) - b \times \alpha(\alpha_0, \rho, C, F)\,\gamma(C)\,\frac{1}{F} - c \times \alpha(\alpha_0, \rho, C, F)\,\gamma(C)\,\frac{1}{F^2}\,\frac{1}{V(F)}, \tag{5}$$

which is solved for F and allows us to compute the fitness cost $\Delta F = F_r - F$.

To estimate the coefficients a, b and c, we fit the model to all experimental data by score maximization. The statistical score S is defined as:

$$S \;=\; -\sum_{k=1}^{N_d} \frac{(\Delta F(\mathbf{x}_k) - \Delta F_k)^2}{2\sigma_k^2}, \tag{6}$$

where $\mathbf{x}_k \equiv (\alpha_0, \rho, C)_k$ is the triplet of genetic and environment variables at data point k, α_0 and ρ being estimated for each strain (see Materials and Methods of the main text) and the external IPTG concentration C being experimentally fixed; $\Delta F(\mathbf{x})$ is the theoretical value of the fitness cost at point $\mathbf{x}$; and ΔF_k and σ_k^2 are the measured fitness cost mean and variance at point k. There are $N_d = 130$ measurement points.

The highest score, $S_{\max} = -417$, is obtained for $a = 0.21$, $b = 0.0026$ and $c = 0.17$. However, it decreases slowly in one direction of the (b, c) plane, suggesting that this fit cannot definitively rule out some contribution of direct cost of transport (see Figure S10). However, as can be seen in Figure S10, c is significantly larger than 0.

Data and the result of the fit are shown in Figure S11 and Figure S1. Only the stable solution was used for the fits (see below the discussion of stability). Figure S1 shows the fitness of the wild type and four mutants at various external IPTG concentrations and the corresponding theoretical curve. Fitness decreases rapidly with IPTG concentration and saturates around 300 μM, well beyond the point of full induction, as previously observed [15]. The model is able to capture the general behavior without any further fitting. The fitness of the strain T275 seems to saturate earlier than the model predicts. This could stem form uncertainty on the parameter K_p[2]. It is also possible that at these low growth rates and high internal inducer concentrations, efflux via LacY and other processes start playing a role.

The score also allows for a quantitative comparison of models. We find a best score of -704 for a model *without* the growth effects described in Section II B. Taking these effects into account thus highly improves the agreement of the model with data.

---

[2] The half-saturation constant K_p of LacY strongly depends on the transported molecule, the strain and other conditions [5, 6, 10], and was not measured for IPTG: we followed a consensus in considering $K_p = 420$ μM [16], but note its arbitrariness.

D. Dynamical analysis

Equation 1 of the main text is a model of the stationary fitness, but it can also be seen as an indication of the way the population will converge toward its steady growth. The effect of a change in IPTG concentration would not have an immediate impact on the growth rate. If we assume that the typical time scale is of the order of one cell cycle, as in particular is induced by dilution, we can rewrite Equation 1 of the main text as a discrete update process:

$$F_{n+1} \;=\; H(F_n), \tag{7}$$

where n is the number of generations and $H(F) := F_r - a\,\alpha - b\,\gamma\,\alpha/F - c\,\gamma\,\alpha/F^2$. Solving Equation 7 when n goes to infinity gives back the steady-state solutions. But we can now also study the *stability* of these solutions. Indeed, if $H(F + \delta F)$ is lower than F upon a perturbation δF around a steady state, then the growth rate will decrease back toward this steady state; that state would thus be *stable*. Inversely, if it is larger than F, then growth rate will keep increasing away from the steady-state, meaning that the solution is *unstable*. The stability of the solutions can be read graphically by plotting $H(F)$, as shown in Figure S3A.

We find generically two positive solutions for the growth rate, the largest being stable, the lowest unstable (see Figure S3A). For some parameter values, there are no solutions. Figure S3A shows that then $H(F) < F$ for all values of F. This means that the growth rate decreases until it reaches its minimum value, zero. We see here how a vicious circle leads to population extinction: cells do not grow fast enough to dilute a toxic product, thus reinforcing its toxicity, and leading to even slower growth. Cells fall in this regime for parameters beyond the "cliff" shown in Figures 4 and 5 of the main text.

We can now also represent the fitness cost ΔF "as a function" of the rate of protein synthesis α and the protein concentration C_z. These two quantities depend on the growth rate. Thus they also vary as the population goes toward its steady state, and one has to be careful while interpreting the dependence of the fitness cost ΔF on α and C_z (see Figure S4). We also see how two different strains, having different initial conditions upon the addition of IPTG, can have the same phenotype α but two different steady-state fitnesses (see Figure S4A, in the range 0.2 to 0.3 of α).

Not only the stability of the steady states, but also the whole time evolution of the population can be read from Equation 7. Indeed, iterating from the initial time $n = 0$ (when for example cells were transferred to a growth medium containing IPTG) leads to:

$$F_n \;=\; H^{(n)}(F_{n=0}). \tag{8}$$

Considering that one time step is one generation, we can translate this evolution in "objective" time $t = n \times \ln 2/F_n$. The result is shown on Figure S3B. The steady state seems to be indeed reached during the experiments. Moreover, one can consider a continuous time process, under the assumption that F varies on a time scale longer than one cell cycle:

$$F_{n+1} - F_n \;=\; F_{t+\ln 2/F_t} - F_t \;\approx\; \dot{F}_t \times \frac{\ln 2}{F_t}. \tag{9}$$

This leads to:

$$\dot{F}_t = \Big(H(F_t) - F_t\Big) F_t \ln 2. \tag{10}$$

Figure S3B shows the time evolutions thus obtained, for various initial growth rates. The stability of the first solution and the instability of the second are clearly visible. This second solution is a threshold of initial growth rate, below which the cells enter the vicious circle described above, and go to extinction.

Overall, the continuous time description matches well the discrete update process. However, both the discrete time description and the approximation leading to Equation 9 break down as F approaches zero and the generation time becomes larger and larger. Here a more careful approach is needed to show the population go asymptotically to extinction.

III. GENOTYPE-ENVIRONMENT INTERACTIONS

Figure S2 shows plots of the fitness cost ΔF, as a function of α_0 and ρ for fixed external IPTG concentration $C =$ 0, 30 and 1000 μM (Figure S2A, B and C), and as a function of C and α_0 for fixed $\rho = 0$, 0.32 and 1 (Figure S2D, E and F).

The form of the fitness landscape (drawn as a function of α_0 and ρ) dramatically depends on the external inducer concentration, another evidence of strong genotype-environment interactions. The shape of the surface on panels D, E and F of Figure S2 is reminiscent of the recently inferred growth landscape of yeast in presence of glucose [17]. A growth feedback is expected to be at play in this system as well, and could thus explain the shape of its growth landscape.

IV. ERROR PROPAGATION

The uncertainties on α_0 and ρ are obtained by assuming independent experimental errors on F and C_z:

$$\delta\alpha_0 = \alpha_0 \times \sqrt{\left(\frac{\delta C_z(C_1,F_1)}{C_z(C_1,F_1)}\right)^2 + \left(\frac{\delta F_1}{4F_0 - F_1}\right)^2},$$

$$\delta\rho = \rho \times \sqrt{\left(\frac{\delta C_z(0,F_\emptyset)}{C_z(0,F_\emptyset)}\right)^2 + \left(\frac{\delta F_\emptyset}{4F_0 - F_\emptyset}\right)^2 + \left(\frac{\delta\alpha_0}{\alpha_0}\right)^2},$$

where δF and δC_z are the measured standard errors of the mean of F and C_z. They are then propagated to evaluate the uncertainty $\delta\alpha$ on the phenotype α:

$$\delta\alpha = \sqrt{\sum_{x=\alpha_0,\rho,F}\left(\frac{\partial\alpha}{\partial x}\delta x\right)^2}.$$

A possible error on the IPTG concentration C is not considered as it is expected to be small.

---

[1] K J Andrews and G D Hegeman, "Selective disadvantage of non-functional protein synthesis in *Escherichia coli*," J Mol Evol **8**, 317–328 (1976).
[2] Bengt v. Hofsten, "The inhibitory effect of galactosides on the growth of *Escherichia coli*," Biochimica et Biophysica Acta **48**, 164–171 (1961).
[3] Erez Dekel and Uri Alon, "Optimality and evolutionary tuning of the expression level of a protein," Nature **436**, 588–592 (2005).
[4] Thomas Kuhlman, Zhongge Zhang, Milton H Saier, and Terence Hwa, "Combinatorial transcriptional control of the lactose operon of *Escherichia coli*," Proc Natl Acad Sci U S A **104**, 6043–6048 (2007).
[5] Adam Kepes, "Études cintétiques sur la galactoside-perméase d'*Escherichia coli*," Biochim Biophys Acta **40**, 70–84 (1960).
[6] Peter C Maloney and T Hastings Wilson, "Quantitative aspects of active transport by the lactose transport system of *Escherichia coli*," Biochim Biophys Acta (BBA) - Biomembranes **330**, 196–205 (1973).
[7] K A Datsenko and B L Wanner, "One-step inactivation of chromosomal genes in *Escherichia coli* K-12 using PCR products," Proc Natl Acad Sci U S A **97**, 6640–6645 (2000).
[8] Anthony M Dean, "Selection and neutrality in lactose operons of *Escherichia coli*," Genetics **123**, 441–454 (1989).
[9] Jason T Noel, Sergei S Pilyugin, and Atul Narang, "The diffusive influx and carrier efflux have a strong effect on the bistability of the *lac* operon in *Escherichia coli*," J Theor Biol **256**, 14–28 (2009).
[10] H H Winkler and T H Wilson, "Inhibition of β-galactoside transport by substrates of the glucose transport system in *Escherichia coli*," Biochimica et Biophysica Acta (BBA)-Biomembranes **135**, 1030–1051 (1967).
[11] Dorothy M Wilson, Resha M Putzrath, and T Hastings Wilson, "Inhibition of growth of *Escherichia coli* by lactose and other galactosides," Biochim Biophys Acta (BBA) - Biomembranes **649**, 377–384 (1981).
[12] Stefan Klumpp, Zhongge Zhang, and Terence Hwa, "Growth rate-dependent global effects on gene expression in bacteria," Cell **139**, 1366–1375 (2009).
[13] Matthew Scott, Carl W Gunderson, Eduard M Mateescu, Zhongge Zhang, and Terence Hwa, "Interdependence of Cell Growth and Gene Expression: Origins and Consequences," Science **330**, 1099–1102 (2010).
[14] M Schaechter, O Maaloe, and N O Kjeldgaard, "Dependency on medium and temperature of cell size and chemical composition during balanced growth of *Salmonella typhimurium*," J Gen Microbiol **19**, 592–606 (1958).
[15] Aaron Novick and Milton Weiner, "Enzyme induction as an all-or-none phenomenon," Proc Natl Acad Sci U S A **43**, 553–566 (1957).
[16] B Cheng, R L Fournier, P A Relue, and J Schisler, "An experimental and theoretical study of the inhibition of *Escherichia coli lac* operon gene expression by antigene oligonucleotides," Biotechnology and bioengineering **74**, 220–229 (2001).
[17] Hyun Youk and Alexander van Oudenaarden, "Growth landscape formed by perception and import of glucose in yeast," Nature **462**, 875–879 (2009).
